# Supplementary material for: Polyketide synthase gene clusters in the ericoid mycorrhizal fungus Oidiodendron maius: genome-wide prediction, expression profiling, and functional characterization of knockout mutants
Source: Front Plant Sci. 2026 Sep 16;17:1924430. doi: 10.3389/fpls.2026.1924430 (PMC13623903; doi:10.3389/fpls.2026.1924430)
Supplement: Supplementary file 1 [file SupplementaryFile1.zip › Supplementary Materials/Supplementary Folder 1.zip!/Oidma1_AssemblyScaffolds_Repeatmasked/knownclusterblast/cluster41/jgi.p_Oidma1_65103_mibig_hits.html]

| MIBiG Protein | Description | MIBiG Cluster | MiBiG Product | % ID | % Coverage | BLAST Score | E-value |
| --- | --- | --- | --- | --- | --- | --- | --- |
| ADM34138.1 | non-ribosomal\_peptide\_synthetase | BGC0001084 | Notoamide\_/\_stephacidin\_biosynthetic\_gene\_cluster | 42 | 101.424332344 | 1308 | 0.0 |
| AGC83576.1 | NRPS | BGC0000818 | Notoamide\_biosynthetic\_gene\_cluster | 42 | 101.424332344 | 1302 | 0.0 |
| BAH23995.1 | nonribosomal\_peptide\_synthetase | BGC0000356 | Fumitremorgin\_biosynthetic\_gene\_cluster | 41 | 101.839762611 | 1261 | 0.0 |
| EFY94500.1 | non-ribosomal\_peptide\_synthetase | BGC0000337 | Destruxins\_biosynthetic\_gene\_cluster | 38 | 101.78041543 | 1131 | 0.0 |
| EAL89049.1 | nonribosomal\_peptide\_synthase,\_putative | BGC0000355 | Fumiquinazolines\_biosynthetic\_gene\_cluster | 38 | 101.246290801 | 1113 | 0.0 |
| ctg1\_orf003 |  | BGC0000334 | Cyclosporin\_biosynthetic\_gene\_cluster | 38 | 101.839762611 | 1079 | 0.0 |
| EAW16180.1 | nonribosomal\_peptide\_synthase,\_putative | BGC0000293 | Acetylaszonalenin\_biosynthetic\_gene\_cluster | 37 | 101.246290801 | 1077 | 0.0 |
| OAQ83772.1 | nonribosomal\_peptide\_synthase | BGC0001358 | Leucinostatins\_biosynthetic\_gene\_cluster | 37 | 104.866468843 | 1077 | 0.0 |
| CBF87069.1 | nonribosomal\_peptide\_synthase,\_putative\_(Eurofung) | BGC0001290 | Emericellamide\_A\_/\_emericellamide\_B\_biosynthetic\_gene\_cluster | 35 | 102.492581602 | 1032 | 4.2e-301 |
| ADY16697.1 | TqaA | BGC0001142 | Tryptoquialanine\_biosynthetic\_gene\_cluster | 36 | 103.857566766 | 1025 | 8.8e-299 |
| CAP96445.1 |  | BGC0000420 | Roquefortine\_C\_/\_meleagrin\_biosynthetic\_gene\_cluster | 36 | 101.721068249 | 1010 | 1.7e-294 |
| BAO73252.1 | non-ribosomal\_peptide\_synthetase | BGC0000456 | Verlamelin\_biosynthetic\_gene\_cluster | 35 | 103.204747774 | 1007 | 1.5e-293 |
| AFT91378.1 | EcdA | BGC0000338 | Echinocandin\_B\_biosynthetic\_gene\_cluster | 35 | 103.264094955 | 1004 | 1.2e-292 |
| EPE34341.1 | Acetyl-CoA\_synthetase-like\_protein | BGC0001035 | Pneumocandin\_biosynthetic\_gene\_cluster | 35 | 102.611275964 | 1002 | 8e-292 |
| BAN91483.1 | putative\_Aculeacin\_A\_nonribosomal\_peptide\_synthase | BGC0001220 | Aculeacin\_A\_biosynthetic\_gene\_cluster | 36 | 102.96735905 | 999 | 4e-291 |
| AMM63162.1 | AniA | BGC0001371 | Echinocandin\_B\_biosynthetic\_gene\_cluster | 35 | 102.96735905 | 997 | 1.5e-290 |
| ABB90279.1 | non-ribosomal\_peptide\_synthetase | BGC0001057 | Zearalenone\_biosynthetic\_gene\_cluster | 35 | 101.958456973 | 986 | 3.5e-287 |
| CAP93139.1 |  | BGC0000357 | Fungisporin\_biosynthetic\_gene\_cluster | 34 | 103.382789318 | 975 | 1.1e-283 |
| AAF01762.1 | AM-toxin\_synthetase | BGC0001261 | AM-toxin\_biosynthetic\_gene\_cluster | 36 | 102.848664688 | 974 | 1.8e-283 |
| AGA37267.1 | NRPS | BGC0000816 | Malbrancheamide\_biosynthetic\_gene\_cluster | 32 | 101.899109792 | 923 | 3.6e-268 |
| XP\_001227984.1 | hypothetical\_protein | BGC0001240 | Serinocyclin\_A\_/\_serinocyclin\_B\_biosynthetic\_gene\_cluster | 37 | 93.234421365 | 895 | 1.1e-259 |
| CRG85572.1 | nonribosomal\_peptide\_synthase,\_putative | BGC0001402 | Cyclochlorotine\_biosynthetic\_gene\_cluster | 33 | 102.848664688 | 888 | 1.3e-257 |
| AGA37269.1 | NRPS | BGC0000819 | Paraherquamide\_biosynthetic\_gene\_cluster | 32 | 97.6854599407 | 847 | 2.5e-245 |
| ACZ66258.1 | APS1 | BGC0000304 | Apicidin\_biosynthetic\_gene\_cluster | 32 | 107.952522255 | 832 | 6.4e-241 |
| ABM91454.1 | lysergyl\_peptide\_synthetase\_LpsB | BGC0000348 | Ergovaline\_biosynthetic\_gene\_cluster | 40 | 64.2729970326 | 780 | 2.9e-225 |
| AGQ43600.1 | HC-toxin\_synthetase | BGC0001166 | HC-toxin\_biosynthetic\_gene\_cluster | 30 | 106.290801187 | 768 | 1.9e-221 |
| AAA33023.2 | HC-toxin\_synthetase | BGC0000370 | HC-toxin\_biosynthetic\_gene\_cluster | 30 | 106.231454006 | 748 | 2.1e-215 |
| ctg1\_orf000000 |  | BGC0000901 | Ferrichrome\_biosynthetic\_gene\_cluster | 38 | 63.5014836795 | 651 | 2e-186 |
| AFN69082.1 | nonribosomal\_peptide\_synthetase\_6 | BGC0001249 | Dimethylcoprogen\_biosynthetic\_gene\_cluster | 36 | 64.4510385757 | 676 | 5.9e-194 |
| AFP87549.1 | NrpS | BGC0001135 | Bicornutin\_biosynthetic\_gene\_cluster | 30 | 104.213649852 | 606 | 1.3e-172 |
| AHZ34233.1 | CifB | BGC0000323 | Cichofactin\_biosynthetic\_gene\_cluster | 28 | 103.738872404 | 581 | 4.4e-165 |
| CBF76038.1 | nonribosomal\_peptide\_synthase,\_putative\_(Eurofung) | BGC0001399 | Fellutamide\_B\_biosynthetic\_gene\_cluster | 34 | 64.9851632047 | 573 | 9.2e-163 |
| AFH75321.1 | nonribosomal\_peptide\_synthetase | BGC0000425 | Sessilin\_biosynthetic\_gene\_cluster | 28 | 103.738872404 | 572 | 1.2e-162 |
| CCJ67640.1 | TaaE | BGC0000447 | Tolaasin\_biosynthetic\_gene\_cluster | 29 | 104.272997033 | 570 | 6e-162 |
| AFH75322.1 | nonribosomal\_peptide\_synthetase | BGC0000425 | Sessilin\_biosynthetic\_gene\_cluster | 29 | 105.28189911 | 570 | 6e-162 |
| KPN93063.1 | NupA | BGC0001416 | Nunapeptin\_/\_nunamycin\_biosynthetic\_gene\_cluster | 28 | 104.09495549 | 567 | 5.1e-161 |
| CBJ90082.1 | Non\_Ribosomal\_peptide\_synthetase\_(-succinylbenzoate--CoA\_ligase) | BGC0001132 | Xenotetrapeptide\_biosynthetic\_gene\_cluster | 29 | 103.857566766 | 566 | 1.5e-160 |
| CAI59267.1 | peptide\_synthetase\_3 | BGC0001263\_c2 | Ergometrine\_biosynthetic\_gene\_cluster | 34 | 63.4421364985 | 561 | 3.6e-159 |
| BAH43870.1 | putative\_linear\_pentadecapeptide\_gramicidin\_synthetase\_LgrB | BGC0000367 | Gramicidin\_biosynthetic\_gene\_cluster | 28 | 105.519287834 | 559 | 1.4e-158 |
| AAO56329.1 | non-ribosomal\_peptide\_synthetase\_SyfB | BGC0000435 | Syringafactin\_biosynthetic\_gene\_cluster | 28 | 103.442136499 | 558 | 3.1e-158 |
| BAH43765.1 | tyrocidine\_synthetase\_II | BGC0000452 | Tyrocidine\_biosynthetic\_gene\_cluster | 27 | 103.857566766 | 556 | 8.9e-158 |
| AIW82283.1 | PuwF | BGC0001125 | Puwainaphycins\_biosynthetic\_gene\_cluster | 27 | 104.866468843 | 553 | 7.5e-157 |
| ACZ55942.1 | non-ribosomal\_peptide\_synthetase | BGC0000302 | Anabaenopeptin\_biosynthetic\_gene\_cluster | 28 | 105.756676558 | 551 | 2.9e-156 |
| BAC67535.1 | arthrofactin\_synthetase\_B | BGC0000305 | Arthrofactin\_biosynthetic\_gene\_cluster | 28 | 104.747774481 | 550 | 4.9e-156 |
| CCJ67639.1 | TaaD | BGC0000447 | Tolaasin\_biosynthetic\_gene\_cluster | 29 | 104.6884273 | 549 | 1.9e-155 |
| AHZ34232.1 | CifA | BGC0000323 | Cichofactin\_biosynthetic\_gene\_cluster | 29 | 104.569732938 | 544 | 4.6e-154 |
| AAY37653.1 | Amino\_acid\_adenylation | BGC0000437 | Syringomycin\_biosynthetic\_gene\_cluster | 27 | 104.035608309 | 544 | 6e-154 |
| AEU11003.1 | NpnC | BGC0001029 | Nostophycin\_biosynthetic\_gene\_cluster | 27 | 104.213649852 | 543 | 1e-153 |
| KPN90376.1 | NupC | BGC0001416 | Nunapeptin\_/\_nunamycin\_biosynthetic\_gene\_cluster | 28 | 104.807121662 | 542 | 2.3e-153 |
| CAD17792.1 | probable\_non\_ribosomal\_peptide\_synthetase\_protein | BGC0001363 | Ralsolamycin\_biosynthetic\_gene\_cluster | 28 | 103.976261128 | 540 | 5.1e-153 |
| ABI26078.1 | OciB | BGC0000331 | Cyanopeptin\_biosynthetic\_gene\_cluster | 27 | 105.341246291 | 540 | 8.6e-153 |
| CAA79245.2 | enniatin\_synthetase | BGC0000342 | Enniatin\_biosynthetic\_gene\_cluster | 29 | 92.8783382789 | 539 | 1.5e-152 |
| BAH43871.1 | truncated\_linear\_pentadecapeptide\_gramicidin\_synthetase\_LgrC | BGC0000367 | Gramicidin\_biosynthetic\_gene\_cluster | 28 | 104.035608309 | 538 | 3.3e-152 |
| AAZ03552.1 | McnC | BGC0000332 | Cyanopeptolin\_biosynthetic\_gene\_cluster | 30 | 92.8189910979 | 537 | 4.3e-152 |
| AFH75320.1 | nonribosomal\_peptide\_synthetase | BGC0000425 | Sessilin\_biosynthetic\_gene\_cluster | 28 | 105.163204748 | 537 | 7.3e-152 |
| AAY37647.1 | Amino\_acid\_adenylation | BGC0000437 | Syringomycin\_biosynthetic\_gene\_cluster | 28 | 105.28189911 | 537 | 7.3e-152 |
| AAO56328.1 | non-ribosomal\_peptide\_synthetase\_SyfA | BGC0000435 | Syringafactin\_biosynthetic\_gene\_cluster | 29 | 105.28189911 | 535 | 2.1e-151 |
| AAY37655.1 | Amino\_acid\_adenylation | BGC0000437 | Syringomycin\_biosynthetic\_gene\_cluster | 27 | 103.560830861 | 534 | 4.7e-151 |
| AAO72425.1 | syringopeptin\_synthetase\_C | BGC0000438 | Syringopeptin\_biosynthetic\_gene\_cluster | 27 | 103.679525223 | 533 | 8.1e-151 |
| BAH22764.1 | nonribosomal\_peptide\_synthetase | BGC0001018 | Micropeptin\_biosynthetic\_gene\_cluster | 28 | 105.816023739 | 532 | 1.8e-150 |
| AGM16412.1 | paenibacterin\_synthetase\_A | BGC0000400 | Paenibacterin\_biosynthetic\_gene\_cluster | 26 | 104.747774481 | 527 | 5.8e-149 |
| ACI30655.1 | BEAS\_beauvericin\_nonribosomal\_cyclodepsipeptide\_synthetase | BGC0000313 | Beauvericin\_biosynthetic\_gene\_cluster | 29 | 96.4985163205 | 527 | 5.8e-149 |
| ADY16689.1 | TqaB | BGC0001142 | Tryptoquialanine\_biosynthetic\_gene\_cluster | 35 | 57.8041543027 | 527 | 7.6e-149 |
| AFH75329.1 | nonribosomal\_peptide\_synthetase | BGC0000398 | Orfamide\_biosynthetic\_gene\_cluster | 28 | 104.629080119 | 527 | 7.6e-149 |
| AED90002.1 | non-ribosomal\_peptide\_synthetase\_ThaA | BGC0000443 | Thanamycin\_biosynthetic\_gene\_cluster | 28 | 105.222551929 | 526 | 9.9e-149 |
| AFJ23825.1 | WLIP\_synthetase\_B | BGC0000462\_c2 | WLIP\_biosynthetic\_gene\_cluster | 28 | 104.213649852 | 526 | 9.9e-149 |
| AGM16414.1 | paenibacterin\_synthetase\_C | BGC0000400 | Paenibacterin\_biosynthetic\_gene\_cluster | 27 | 104.569732938 | 525 | 2.2e-148 |
| AGM14933.1 | xantholysin\_synthetase\_B | BGC0000463 | Xantholysin\_biosynthetic\_gene\_cluster | 28 | 104.154302671 | 524 | 3.8e-148 |
| AAY91421.3 | non-ribosomal\_peptide\_synthetase\_OfaC | BGC0000399 | Orfamide\_biosynthetic\_gene\_cluster | 27 | 104.154302671 | 524 | 4.9e-148 |
| EAL89046.1 | nonribosomal\_peptide\_synthase,\_putative | BGC0000355 | Fumiquinazolines\_biosynthetic\_gene\_cluster | 35 | 59.4065281899 | 523 | 1.1e-147 |
| CAY48788.1 | putative\_non-ribosomal\_peptide\_synthetase | BGC0001312 | Viscosin\_biosynthetic\_gene\_cluster | 28 | 103.798219585 | 522 | 1.9e-147 |
| AAC06348.1 | bacitracin\_synthetase\_3 | BGC0000310 | Bacitracin\_biosynthetic\_gene\_cluster | 27 | 103.264094955 | 521 | 3.2e-147 |
| AHZ34239.1 | CipB | BGC0001389 | Cichopeptin\_biosynthetic\_gene\_cluster | 28 | 104.035608309 | 520 | 7.1e-147 |
| ABI26077.1 | OciA | BGC0000331 | Cyanopeptin\_biosynthetic\_gene\_cluster | 27 | 105.578635015 | 520 | 9.2e-147 |
| AHZ34243.1 | CipF | BGC0001389 | Cichopeptin\_biosynthetic\_gene\_cluster | 27 | 105.222551929 | 519 | 1.6e-146 |
| AAO23333.1 | NcpA | BGC0000397 | Nostocyclopeptide\_biosynthetic\_gene\_cluster | 28 | 104.391691395 | 518 | 2.7e-146 |
| CCJ67636.1 | TaaA | BGC0000447 | Tolaasin\_biosynthetic\_gene\_cluster | 27 | 105.163204748 | 518 | 2.7e-146 |
| ANQ43347.1 | non-ribosomal\_peptide\_synthetase | BGC0001347 | Poaeamide\_B\_biosynthetic\_gene\_cluster | 27 | 102.789317507 | 518 | 3.5e-146 |
| AIW82284.1 | PuwG | BGC0001125 | Puwainaphycins\_biosynthetic\_gene\_cluster | 27 | 106.528189911 | 518 | 3.5e-146 |
| CDG17987.1 | Putative\_Ornithine\_racemase\_(fragment) | BGC0000464 | Xenoamicins\_biosynthetic\_gene\_cluster | 27 | 106.765578635 | 517 | 4.6e-146 |
| ABV79985.1 | ApnA | BGC0000301 | Anabaenopeptin\_biosynthetic\_gene\_cluster | 27 | 106.528189911 | 517 | 7.8e-146 |
| EFE73313.1 | nonribosomal\_peptide\_synthetase | BGC0000431 | Stenothricin\_biosynthetic\_gene\_cluster | 28 | 104.510385757 | 515 | 1.7e-145 |
| BAC67536.1 | arthrofactin\_synthetase\_C | BGC0000305 | Arthrofactin\_biosynthetic\_gene\_cluster | 27 | 105.400593472 | 515 | 2.3e-145 |
| AAY37654.1 | Amino\_acid\_adenylation | BGC0000437 | Syringomycin\_biosynthetic\_gene\_cluster | 27 | 104.09495549 | 515 | 3e-145 |
| CDG17980.1 | Putative\_Ornithine\_racemase\_(fragment) | BGC0000464 | Xenoamicins\_biosynthetic\_gene\_cluster | 27 | 105.994065282 | 514 | 3.9e-145 |
| CAQ71829.1 | non\_ribosomal\_peptide\_synthase,\_antibiotic\_synthesis;\_contains\_4\_condensation\_domains,\_3\_AMP-acid\_ligases\_II\_domains,\_3\_PP-binding,\_Phosphopantetheine\_attachment\_site\_and\_a\_putative\_thioesterase\_domain | BGC0001189 | Taiwachelin\_biosynthetic\_gene\_cluster | 28 | 104.6884273 | 514 | 5.1e-145 |
| AAU39359.1 | lichenysin\_synthase\_LchAA | BGC0000381 | Lichenysin\_biosynthetic\_gene\_cluster | 26 | 103.916913947 | 514 | 6.6e-145 |
| AGS77307.1 | NRPS\_modules\_1-2 | BGC0001178 | UK-68,597\_biosynthetic\_gene\_cluster | 27 | 102.670623145 | 514 | 6.6e-145 |
| KPN93064.1 | NupB | BGC0001416 | Nunapeptin\_/\_nunamycin\_biosynthetic\_gene\_cluster | 27 | 104.035608309 | 513 | 1.1e-144 |
| CAD17793.1 | probable\_non\_ribosomal\_peptide\_synthetase\_protein | BGC0001363 | Ralsolamycin\_biosynthetic\_gene\_cluster | 28 | 104.510385757 | 512 | 2.5e-144 |
| ACJ04424.1 | aureobasidin\_A1\_biosynthesis\_complex | BGC0000307 | Aureobasidin\_A1\_biosynthetic\_gene\_cluster | 28 | 95.4896142433 | 511 | 4.3e-144 |
| ACR78148.1 | BSLS | BGC0000312 | Bassianolide\_biosynthetic\_gene\_cluster | 31 | 63.7982195846 | 511 | 4.3e-144 |
| AAU39360.1 | lichenysin\_synthase\_LchAB | BGC0000381 | Lichenysin\_biosynthetic\_gene\_cluster | 26 | 104.272997033 | 510 | 5.6e-144 |
| CAE02631.1 | surfactin\_synthetase\_B | BGC0000433 | Surfactin\_biosynthetic\_gene\_cluster | 26 | 103.560830861 | 510 | 5.6e-144 |
| KPN90369.1 | NunE | BGC0001416 | Nunapeptin\_/\_nunamycin\_biosynthetic\_gene\_cluster | 28 | 106.409495549 | 510 | 7.3e-144 |
| ABH06368.2 | MassB | BGC0000389 | Massetolide\_A\_biosynthetic\_gene\_cluster | 27 | 104.272997033 | 509 | 1.2e-143 |
| AAY91420.2 | non-ribosomal\_peptide\_synthetase\_OfaB | BGC0000399 | Orfamide\_biosynthetic\_gene\_cluster | 27 | 104.154302671 | 508 | 2.8e-143 |
| AED90003.1 | non-ribosomal\_peptide\_synthetase\_ThaB | BGC0000443 | Thanamycin\_biosynthetic\_gene\_cluster | 28 | 105.400593472 | 507 | 8.1e-143 |
| CAE53350.1 | non-ribosomal\_peptide\_synthetase | BGC0000440 | Teicoplanin\_biosynthetic\_gene\_cluster | 28 | 103.026706231 | 506 | 1.1e-142 |
| CAG15009.1 | peptide\_synthetase,\_module\_1-2 | BGC0000441 | Teicoplanin\_biosynthetic\_gene\_cluster | 28 | 103.026706231 | 505 | 1.8e-142 |
| ANQ43349.1 | non-ribosomal\_peptide\_synthetase | BGC0001347\_c2 | Poaeamide\_B\_biosynthetic\_gene\_cluster | 27 | 103.857566766 | 504 | 4e-142 |
| AAO72424.1 | syringopeptin\_synthetase\_B | BGC0000438 | Syringopeptin\_biosynthetic\_gene\_cluster | 27 | 103.916913947 | 504 | 5.3e-142 |
| ABD65958.1 | nonribosomal\_peptide\_synthetase | BGC0000341 | Enduracidin\_biosynthetic\_gene\_cluster | 28 | 103.679525223 | 503 | 6.9e-142 |
| CCJ67637.1 | TaaB | BGC0000447 | Tolaasin\_biosynthetic\_gene\_cluster | 28 | 104.866468843 | 503 | 6.9e-142 |
| AHZ34242.1 | CipE | BGC0001389 | Cichopeptin\_biosynthetic\_gene\_cluster | 27 | 103.442136499 | 503 | 9e-142 |
| AEI58865.1 | peptide\_synthetase | BGC0000455 | Vancomycin\_biosynthetic\_gene\_cluster | 29 | 102.848664688 | 502 | 2.6e-141 |
| WP\_020635019.1 | hypothetical\_protein | BGC0001211 | Albachelin\_biosynthetic\_gene\_cluster | 29 | 90.4451038576 | 501 | 3.4e-141 |
| AGM16413.1 | paenibacterin\_synthetase\_B | BGC0000400 | Paenibacterin\_biosynthetic\_gene\_cluster | 26 | 104.213649852 | 501 | 3.4e-141 |
| CAA11794.1 | PCZA363.3 | BGC0000322 | Chloroeremomycin\_biosynthetic\_gene\_cluster | 28 | 102.611275964 | 501 | 4.4e-141 |
| ABS74208.1 | FenB | BGC0001095 | Fengycin\_biosynthetic\_gene\_cluster | 26 | 104.629080119 | 500 | 5.8e-141 |
| ctg1\_orf00001 |  | BGC0000901 | Ferrichrome\_biosynthetic\_gene\_cluster | 33 | 58.9910979228 | 499 | 1.3e-140 |
| AGM14934.1 | xantholysin\_synthetase\_C | BGC0000463 | Xantholysin\_biosynthetic\_gene\_cluster | 27 | 103.620178042 | 499 | 1.7e-140 |
| AGE25480.1 | amino\_acid\_adenylation\_protein | BGC0001208\_c2 | Poaeamide\_biosynthetic\_gene\_cluster | 27 | 102.96735905 | 498 | 2.9e-140 |
| AHI59109.1 | locillomycin\_synthase\_B | BGC0001005 | Locillomycin\_biosynthetic\_gene\_cluster | 25 | 104.332344214 | 498 | 2.9e-140 |
| KPN93065.1 | NunD | BGC0001416 | Nunapeptin\_/\_nunamycin\_biosynthetic\_gene\_cluster | 27 | 105.697329377 | 498 | 2.9e-140 |
| CDG17985.1 | Putative\_Ornithine\_racemase\_(fragment) | BGC0000464 | Xenoamicins\_biosynthetic\_gene\_cluster | 27 | 105.222551929 | 498 | 2.9e-140 |
| BAH43766.1 | tyrocidine\_synthetase\_III | BGC0000452 | Tyrocidine\_biosynthetic\_gene\_cluster | 26 | 103.916913947 | 498 | 3.8e-140 |
| AFH75328.1 | nonribosomal\_peptide\_synthetase | BGC0000398 | Orfamide\_biosynthetic\_gene\_cluster | 27 | 102.077151335 | 497 | 8.4e-140 |
| ADJ63842.1 | non-ribosomal\_peptide\_synthetase\_protein | BGC0000424 | Serobactins\_biosynthetic\_gene\_cluster | 27 | 104.451038576 | 496 | 1.1e-139 |
| ANQ43348.1 | non-ribosomal\_peptide\_synthetase | BGC0001347\_c2 | Poaeamide\_B\_biosynthetic\_gene\_cluster | 27 | 103.620178042 | 496 | 1.4e-139 |
| AAF99707.2 | syringopeptin\_synthetase | BGC0000438 | Syringopeptin\_biosynthetic\_gene\_cluster | 27 | 104.451038576 | 495 | 3.2e-139 |
| AOA33121.1 | nonribosomal\_peptide\_synthetase | BGC0001346 | Bananamides\_biosynthetic\_gene\_cluster | 27 | 104.272997033 | 493 | 7.1e-139 |
| AHJ31215.1 | Long-chain-fatty-acid--CoA\_ligase | BGC0000430 | Spumigin\_biosynthetic\_gene\_cluster | 27 | 107.299703264 | 491 | 3.5e-138 |
| AEH59100.1 | amino\_acid\_adenylation\_domain-containing\_protein/NRPS | BGC0000385 | Lysobactin\_biosynthetic\_gene\_cluster | 27 | 105.103857567 | 491 | 4.6e-138 |
| AAF17280.1 | NosC | BGC0001028 | Nostopeptolide\_biosynthetic\_gene\_cluster | 27 | 106.765578635 | 491 | 4.6e-138 |
| AEW31020.1 | plipastatin\_synthetase | BGC0000407 | Plipastatin\_biosynthetic\_gene\_cluster | 26 | 104.807121662 | 489 | 1.3e-137 |
| AFJ14794.1 | PlpE | BGC0000403 | Pelgipeptin\_biosynthetic\_gene\_cluster | 26 | 105.222551929 | 489 | 1.7e-137 |
| CCJ67638.1 | TaaC | BGC0000447 | Tolaasin\_biosynthetic\_gene\_cluster | 28 | 105.756676558 | 487 | 5.1e-137 |
| BAH43869.1 | linear\_pentadecapeptide\_gramicidin\_synthetase\_LgrA | BGC0000367 | Gramicidin\_biosynthetic\_gene\_cluster | 27 | 104.6884273 | 487 | 5.1e-137 |
| AAC06347.1 | bacitracin\_synthetase\_2 | BGC0000310 | Bacitracin\_biosynthetic\_gene\_cluster | 26 | 103.50148368 | 487 | 6.6e-137 |
| AAF17281.1 | NosD | BGC0001028 | Nostopeptolide\_biosynthetic\_gene\_cluster | 27 | 106.172106825 | 487 | 8.7e-137 |
| CAJ34381.1 | NRPS\_protein | BGC0000445 | Thiocoraline\_biosynthetic\_gene\_cluster | 27 | 105.400593472 | 486 | 1.1e-136 |
| ACA09733.2 | fusaricidin\_synthetase | BGC0000358 | Fusaricidin\_biosynthetic\_gene\_cluster | 28 | 92.4629080119 | 486 | 1.1e-136 |
| AHZ34241.1 | CipD | BGC0001389 | Cichopeptin\_biosynthetic\_gene\_cluster | 28 | 103.738872404 | 486 | 1.5e-136 |
| CBJ79916.1 | putative\_Ornithine\_racemase | BGC0001133 | Taxlllaid\_biosynthetic\_gene\_cluster | 28 | 101.364985163 | 485 | 2.5e-136 |
| CAK15814.1 | putative\_non-ribosomal\_peptide\_synthetase,\_terminal\_component | BGC0000344 | Entolysin\_biosynthetic\_gene\_cluster | 27 | 105.578635015 | 485 | 2.5e-136 |
| ABF87031.1 | non-ribosomal\_peptide\_synthetase/polyketide\_synthase | BGC0000393 | Myxoprincomide\_biosynthetic\_gene\_cluster | 27 | 106.409495549 | 483 | 9.6e-136 |
| ABD65957.1 | nonribosomal\_peptide\_synthetase | BGC0000341 | Enduracidin\_biosynthetic\_gene\_cluster | 27 | 103.50148368 | 483 | 1.3e-135 |
| AAK81824.1 | peptide\_synthetase | BGC0000326 | Complestatin\_biosynthetic\_gene\_cluster | 28 | 92.6409495549 | 482 | 1.6e-135 |
| CAE02630.1 | surfactin\_synthetase\_A | BGC0000433 | Surfactin\_biosynthetic\_gene\_cluster | 27 | 104.272997033 | 481 | 3.6e-135 |
| ABW17376.1 | PsoB | BGC0000411 | Putisolvin\_biosynthetic\_gene\_cluster | 28 | 91.8100890208 | 480 | 6.2e-135 |
| AFJ23819.1 | WLIP\_synthetase\_A | BGC0000462 | WLIP\_biosynthetic\_gene\_cluster | 27 | 104.332344214 | 478 | 2.4e-134 |
| ABX37382.1 | amino\_acid\_adenylation\_domain\_protein | BGC0000984 | Delftibactin\_biosynthetic\_gene\_cluster | 27 | 105.519287834 | 476 | 1.2e-133 |
| ABS74180.1 | BmyB | BGC0001090 | Bacillomycin\_biosynthetic\_gene\_cluster | 27 | 105.459940653 | 476 | 1.2e-133 |
| AAO23334.1 | NcpB | BGC0000397 | Nostocyclopeptide\_biosynthetic\_gene\_cluster | 27 | 100.474777448 | 476 | 1.2e-133 |
| EFE73312.1 | nonribosomal\_peptide\_synthetase | BGC0000431 | Stenothricin\_biosynthetic\_gene\_cluster | 28 | 105.578635015 | 476 | 1.5e-133 |
| ABW17375.1 | PsoA | BGC0000411 | Putisolvin\_biosynthetic\_gene\_cluster | 27 | 104.510385757 | 475 | 2.6e-133 |
| ABS74206.1 | FenD | BGC0001095 | Fengycin\_biosynthetic\_gene\_cluster | 26 | 104.6884273 | 474 | 5.8e-133 |
| CAK15815.1 | putative\_non\_ribosomal\_peptide\_synthetase | BGC0000344 | Entolysin\_biosynthetic\_gene\_cluster | 28 | 92.6409495549 | 473 | 1.3e-132 |
| AJF34464.1 | Txo2 | BGC0001207 | Teixobactin\_biosynthetic\_gene\_cluster | 26 | 105.044510386 | 472 | 2.9e-132 |
| CAM56770.1 |  | BGC0000354 | Friulimicin\_biosynthetic\_gene\_cluster | 27 | 103.50148368 | 472 | 2.9e-132 |
| AHD05678.1 | nonribosomal\_peptide\_ligase\_subunit | BGC0000402 | Paenilarvins\_biosynthetic\_gene\_cluster | 27 | 105.341246291 | 471 | 4.9e-132 |
| AEH59099.1 | amino\_acid\_adenylation\_domain-containing\_protein/NRPS | BGC0000385 | Lysobactin\_biosynthetic\_gene\_cluster | 27 | 105.459940653 | 470 | 8.4e-132 |
| AEH41794.1 | HrmP | BGC0000374 | Hormaomycin\_biosynthetic\_gene\_cluster | 27 | 104.807121662 | 469 | 1.9e-131 |
| ACM79812.1 | ZmaQ | BGC0001059 | Zwittermycin\_A\_biosynthetic\_gene\_cluster | 25 | 105.163204748 | 468 | 2.4e-131 |
| AAY93445.1 | non-ribosomal\_peptide\_synthetase\_PvdL | BGC0000413 | Pyoverdine\_biosynthetic\_gene\_cluster | 28 | 104.807121662 | 468 | 3.2e-131 |
| ABW17377.1 | PsoC | BGC0000411 | Putisolvin\_biosynthetic\_gene\_cluster | 27 | 103.976261128 | 468 | 4.2e-131 |
| BAB69699.1 | iturin\_A\_synthetase\_B | BGC0001098 | Iturin\_biosynthetic\_gene\_cluster | 26 | 105.222551929 | 468 | 4.2e-131 |
| ACA97576.1 | PmxA | BGC0000408 | Polymyxin\_biosynthetic\_gene\_cluster | 26 | 106.824925816 | 467 | 9.3e-131 |
| AGE26255.1 | putative\_non-ribosomal\_peptide\_synthetase | BGC0001208 | Poaeamide\_biosynthetic\_gene\_cluster | 28 | 92.3442136499 | 466 | 1.2e-130 |
| AFJ23826.1 | WLIP\_synthetase\_C | BGC0000462\_c2 | WLIP\_biosynthetic\_gene\_cluster | 27 | 103.50148368 | 465 | 2.1e-130 |
| ABH06369.2 | MassC | BGC0000389 | Massetolide\_A\_biosynthetic\_gene\_cluster | 28 | 104.154302671 | 463 | 7.9e-130 |
| ABX37383.1 | amino\_acid\_adenylation\_domain\_protein | BGC0000984 | Delftibactin\_biosynthetic\_gene\_cluster | 27 | 105.697329377 | 462 | 1.8e-129 |
| CCJ67648.1 | JagD | BGC0001127 | Jagaricin\_biosynthetic\_gene\_cluster | 27 | 105.756676558 | 460 | 1.1e-128 |
| ALK27916.1 | non-ribosomal\_peptide\_synthase | BGC0001233 | Feglymycin\_biosynthetic\_gene\_cluster | 27 | 104.866468843 | 459 | 1.9e-128 |
| KFH48607.1 | N-like\_protein | BGC0000317 | Cephalosporin\_biosynthetic\_gene\_cluster | 27 | 95.0741839763 | 459 | 1.9e-128 |
| AAG02355.1 | peptide\_synthetase\_NRPS9-8 | BGC0000963 | Bleomycin\_biosynthetic\_gene\_cluster | 28 | 105.816023739 | 457 | 5.6e-128 |
| AHZ20781.1 | non-ribosomal\_peptide\_synthase | BGC0000369 | Hassallidins\_biosynthetic\_gene\_cluster | 26 | 104.154302671 | 456 | 9.6e-128 |
| AJF34463.1 | Txo1 | BGC0001207 | Teixobactin\_biosynthetic\_gene\_cluster | 26 | 105.103857567 | 456 | 9.6e-128 |
| AEZ51520.1 | PmxE | BGC0001153 | Polymyxin\_biosynthetic\_gene\_cluster | 26 | 107.06231454 | 455 | 3.7e-127 |
| AHY86403.1 | non\_ribosomal\_peptide\_synthetase | BGC0000329 | Corpeptin\_biosynthetic\_gene\_cluster | 27 | 99.703264095 | 454 | 6.2e-127 |
| AHZ34240.1 | CipC | BGC0001389 | Cichopeptin\_biosynthetic\_gene\_cluster | 27 | 98.1008902077 | 452 | 1.8e-126 |
| EWS95124.1 | hypothetical\_protein | BGC0000306 | Arylomycin\_biosynthetic\_gene\_cluster | 26 | 104.807121662 | 451 | 3.1e-126 |
| AAZ23078.1 | peptide\_synthetase | BGC0000291 | A54145\_biosynthetic\_gene\_cluster | 28 | 105.519287834 | 445 | 2.2e-124 |
| ABA70582.1 | alpha-aminoadypil-cysteinyl-valine\_synthetase | BGC0000404 | Penicillin\_biosynthetic\_gene\_cluster | 26 | 106.053412463 | 445 | 3.8e-124 |
| CBJ79915.1 | putative\_Phenylalanine\_racemase\_(ATP-hydrolyzing) | BGC0001133 | Taxlllaid\_biosynthetic\_gene\_cluster | 27 | 102.017804154 | 445 | 3.8e-124 |
| ABR12615.1 | ACV\_synthetase | BGC0000405 | Penicillin\_biosynthetic\_gene\_cluster | 26 | 106.053412463 | 444 | 6.4e-124 |
| ACA97580.1 | PmxE | BGC0000408 | Polymyxin\_biosynthetic\_gene\_cluster | 26 | 108.84272997 | 444 | 6.4e-124 |
| AAF08796.1 | MycB | BGC0001103 | Mycosubtilin\_biosynthetic\_gene\_cluster | 26 | 105.519287834 | 443 | 8.4e-124 |
| ALK27914.1 | non-ribosomal\_peptide\_synthase | BGC0001233 | Feglymycin\_biosynthetic\_gene\_cluster | 27 | 106.172106825 | 443 | 1.4e-123 |
| CAB15186.3 | siderophore\_2,3-dihydroxybenzoate-glycine-threonine\_trimeric\_ester\_bacillibactin\_synthetase | BGC0000309 | Bacillibactin\_biosynthetic\_gene\_cluster | 27 | 105.637982196 | 441 | 4.2e-123 |
| CAY48789.1 | putative\_non-ribosomal\_peptide\_synthetase | BGC0001312 | Viscosin\_biosynthetic\_gene\_cluster | 27 | 92.4035608309 | 437 | 6e-122 |
| ACM79806.1 | ZmaB | BGC0001059 | Zwittermycin\_A\_biosynthetic\_gene\_cluster | 25 | 102.848664688 | 437 | 6e-122 |
| AJM89738.1 | PmxE | BGC0001192 | Colistin\_biosynthetic\_gene\_cluster | 25 | 108.902077151 | 436 | 1.8e-121 |
| ABL74936.1 | TlmX | BGC0001048 | Tallysomycin\_biosynthetic\_gene\_cluster | 27 | 104.629080119 | 435 | 2.3e-121 |
| AGN74876.1 | nonribosomal\_peptide\_synthetase | BGC0000459 | Griseoviridin\_/\_viridogrisein\_biosynthetic\_gene\_cluster | 27 | 106.587537092 | 429 | 2.1e-119 |
| ALV82356.1 | CDA\_peptide\_synthetase\_I | BGC0001370 | Lipopeptide\_8D1-1\_/\_lipopeptide\_8D1-2\_biosynthetic\_gene\_cluster | 26 | 104.866468843 | 426 | 1.1e-118 |
| ACG60782.1 | NRPS(C/A/PCP/C/A/PCP) | BGC0001058 | Zorbamycin\_biosynthetic\_gene\_cluster | 26 | 104.272997033 | 414 | 7.1e-115 |
| AJV88375.1 | MfnC | BGC0001214 | Marformycins\_biosynthetic\_gene\_cluster | 26 | 105.459940653 | 413 | 9.3e-115 |
| CAG29032.1 | nonribosomal\_peptide\_synthetase\_(modules\_3\_to\_6) | BGC0001023 | Myxochromide\_biosynthetic\_gene\_cluster | 26 | 103.976261128 | 411 | 4.6e-114 |
| AGC09528.1 | NRPS | BGC0001183 | Lobophorin\_biosynthetic\_gene\_cluster | 26 | 104.985163205 | 410 | 1.3e-113 |
| ADH04679.1 | non-ribosomal\_peptide\_synthetase | BGC0001344 | Tubulysin\_biosynthetic\_gene\_cluster | 27 | 93.056379822 | 409 | 2.3e-113 |
| AET13875.1 | epichloenin\_A\_synthetase | BGC0001250 | Epichloenin\_A\_biosynthetic\_gene\_cluster | 25 | 107.715133531 | 404 | 4.3e-112 |
| AET13879.1 | epichloenin\_A\_synthetase | BGC0001251 | Epichloenin\_A\_biosynthetic\_gene\_cluster | 25 | 107.774480712 | 401 | 3.7e-111 |
| AHF21228.1 | TriD | BGC0000449 | Tridecaptin\_biosynthetic\_gene\_cluster | 29 | 65.6973293769 | 397 | 9e-110 |
| CCM44337.1 | putative\_nonribosomal\_peptide\_synthetase | BGC0001056 | Zeamine\_biosynthetic\_gene\_cluster | 25 | 105.697329377 | 391 | 5e-108 |
| CBZ42146.1 | putative\_non-ribosomal\_peptide\_synthetase | BGC0001117 | Himastatin\_biosynthetic\_gene\_cluster | 29 | 65.0445103858 | 390 | 1.1e-107 |
| CCM44336.1 | putative\_nonribosomal\_peptide\_synthetase | BGC0001056 | Zeamine\_biosynthetic\_gene\_cluster | 25 | 106.290801187 | 390 | 1.1e-107 |
| AEG64697.1 | LpmC | BGC0000379 | Laspartomycin\_biosynthetic\_gene\_cluster | 27 | 92.8783382789 | 389 | 1.9e-107 |
| AAZ23077.1 | peptide\_synthetase | BGC0000291 | A54145\_biosynthetic\_gene\_cluster | 25 | 105.637982196 | 385 | 2.7e-106 |
| ABQ96384.2 | fusaricidin\_synthetase | BGC0001152 | Fusaricidin\_biosynthetic\_gene\_cluster | 28 | 64.4510385757 | 383 | 1.4e-105 |
| CAM02313.1 | putative\_non-ribosomal\_peptide\_synthetase | BGC0000349 | Erythrochelin\_biosynthetic\_gene\_cluster | 26 | 93.175074184 | 382 | 3e-105 |
| AAM80539.1 | StaA | BGC0000290 | A47934\_biosynthetic\_gene\_cluster | 28 | 74.4213649852 | 379 | 2.5e-104 |
| AEU11006.1 | NpnB | BGC0001029 | Nostophycin\_biosynthetic\_gene\_cluster | 30 | 65.1038575668 | 376 | 1.7e-103 |
| BAH22763.1 | nonribosomal\_peptide\_synthetase | BGC0001018 | Micropeptin\_biosynthetic\_gene\_cluster | 28 | 66.46884273 | 373 | 1.1e-102 |
| AIE77057.1 | peptide\_synthetase | BGC0000418 | Ristocetin\_biosynthetic\_gene\_cluster | 27 | 73.7685459941 | 373 | 1.8e-102 |
| AAZ03551.1 | McnB | BGC0000332 | Cyanopeptolin\_biosynthetic\_gene\_cluster | 28 | 66.46884273 | 370 | 9.1e-102 |
| AIG79243.1 | Hypothetical\_protein | BGC0000419 | Ristomycin\_A\_biosynthetic\_gene\_cluster | 27 | 74.0652818991 | 369 | 2e-101 |
| EFL06867.1 | predicted\_protein | BGC0000300 | Amychelin\_biosynthetic\_gene\_cluster | 27 | 95.1928783383 | 360 | 9.4e-99 |
| AEA30273.1 | peptide\_synthetase | BGC0000429 | Skyllamycin\_biosynthetic\_gene\_cluster | 26 | 64.4510385757 | 359 | 1.6e-98 |
| CAC48360.1 | peptide\_synthetase | BGC0000311 | Balhimycin\_biosynthetic\_gene\_cluster | 28 | 74.4807121662 | 359 | 2.1e-98 |
| AAM80537.1 | StaC | BGC0000290 | A47934\_biosynthetic\_gene\_cluster | 28 | 66.409495549 | 358 | 3.6e-98 |
| ACM68690.1 | AerG1 | BGC0000298 | Aeruginosin\_biosynthetic\_gene\_cluster | 28 | 66.1127596439 | 358 | 6.1e-98 |
| ABV79986.1 | ApnB | BGC0000301 | Anabaenopeptin\_biosynthetic\_gene\_cluster | 28 | 65.5192878338 | 357 | 7.9e-98 |
| AAF00960.1 | McyA | BGC0001017 | Microcystin\_biosynthetic\_gene\_cluster | 27 | 74.7181008902 | 357 | 1e-97 |
| AHZ20773.1 | non-ribosomal\_peptide\_synthase | BGC0000369 | Hassallidins\_biosynthetic\_gene\_cluster | 27 | 65.5192878338 | 356 | 1.4e-97 |
| WP\_028678149.1 | non-ribosomal\_peptide\_synthetase | BGC0001228 | Retimycin\_biosynthetic\_gene\_cluster | 29 | 65.0445103858 | 355 | 3e-97 |
| CAD29797.1 | peptide\_synthetase | BGC0001015 | Microcystin\_biosynthetic\_gene\_cluster | 27 | 75.3709198813 | 355 | 3e-97 |
| CAM59606.1 | non-ribosomal\_peptide\_synthetase | BGC0000297 | Aeruginoside\_biosynthetic\_gene\_cluster | 27 | 65.1632047478 | 351 | 4.4e-96 |
| AIW82285.1 | PuwH | BGC0001125 | Puwainaphycins\_biosynthetic\_gene\_cluster | 28 | 65.5192878338 | 350 | 1.3e-95 |
| ALV82388.1 | CDA\_peptide\_synthetase\_III | BGC0001370 | Lipopeptide\_8D1-1\_/\_lipopeptide\_8D1-2\_biosynthetic\_gene\_cluster | 28 | 65.5786350148 | 349 | 1.7e-95 |
| AEI58866.1 | peptide\_synthetase | BGC0000455 | Vancomycin\_biosynthetic\_gene\_cluster | 27 | 64.3323442136 | 349 | 2.2e-95 |
| AFK57212.1 | DidA | BGC0000985 | Didemnin\_biosynthetic\_gene\_cluster | 26 | 103.738872404 | 347 | 8.2e-95 |
| CAE53352.1 | non-ribosomal\_peptide\_synthetase | BGC0000440 | Teicoplanin\_biosynthetic\_gene\_cluster | 28 | 64.6884272997 | 347 | 8.2e-95 |
| CAG15011.1 | peptide\_synthetase,\_module\_4-6 | BGC0000441 | Teicoplanin\_biosynthetic\_gene\_cluster | 28 | 64.6884272997 | 347 | 1.1e-94 |
| ACZ55943.1 | non-ribosomal\_peptide\_synthetase | BGC0000302 | Anabaenopeptin\_biosynthetic\_gene\_cluster | 28 | 65.7566765579 | 346 | 1.8e-94 |
| AET98906.1 | putative\_non-ribosomal\_peptide\_synthetase | BGC0000415 | Quinomycin\_biosynthetic\_gene\_cluster | 28 | 68.6646884273 | 345 | 4.1e-94 |
| CAD55498.1 | CDA\_peptide\_synthetase\_III | BGC0000315 | Calcium-dependent\_antibiotic\_biosynthetic\_gene\_cluster | 27 | 65.8753709199 | 345 | 4.1e-94 |
| AIG79241.1 | Hypothetical\_protein | BGC0000419 | Ristomycin\_A\_biosynthetic\_gene\_cluster | 28 | 64.7477744807 | 344 | 5.3e-94 |
| BAH04162.1 | trsJ | BGC0000450 | Triostin\_A\_biosynthetic\_gene\_cluster | 28 | 68.6053412463 | 342 | 3.5e-93 |
| CAG29031.1 | nonribosomal\_peptide\_synthetase\_(modules\_1\_and\_2) | BGC0001023 | Myxochromide\_biosynthetic\_gene\_cluster | 27 | 75.0741839763 | 341 | 4.5e-93 |
| AIE77059.1 | peptide\_synthetase | BGC0000418 | Ristocetin\_biosynthetic\_gene\_cluster | 28 | 64.7477744807 | 341 | 4.5e-93 |
| AGS77309.1 | NRPS\_modules\_4-6 | BGC0001178 | UK-68,597\_biosynthetic\_gene\_cluster | 27 | 64.3916913947 | 340 | 1.3e-92 |
| AAT01807.1 | non-ribosomal\_peptide\_synthetase | BGC0000365 | Glycopeptidolipid\_biosynthetic\_gene\_cluster | 27 | 65.1038575668 | 338 | 3.8e-92 |
| AEA30274.1 | peptide\_synthetase | BGC0000429 | Skyllamycin\_biosynthetic\_gene\_cluster | 26 | 65.1038575668 | 338 | 3.8e-92 |
| AEZ51516.1 | PmxA | BGC0001153 | Polymyxin\_biosynthetic\_gene\_cluster | 27 | 64.9258160237 | 338 | 5e-92 |
| CAA11795.1 | PCZA363.4 | BGC0000322 | Chloroeremomycin\_biosynthetic\_gene\_cluster | 27 | 64.9851632047 | 337 | 6.5e-92 |
| AAD44234.1 | PstB | BGC0000362 | Glycopeptidolipid\_biosynthetic\_gene\_cluster | 28 | 64.4510385757 | 337 | 8.5e-92 |
| AHD05614.1 | putative\_non-ribosomal\_peptide\_ligase/\_polyketide\_synthase\_hybrid | BGC0001033 | Paenilamicin\_biosynthetic\_gene\_cluster | 27 | 64.5103857567 | 337 | 8.5e-92 |
| EJK79843.1 | amino\_acid\_adenylation\_enzyme/thioester\_reductase\_family\_protein | BGC0000436 | Syringolin\_A\_biosynthetic\_gene\_cluster | 24 | 105.103857567 | 336 | 1.9e-91 |
| CBG75492.1 | putative\_NRPS/siderophore\_biosynthesis\_protein | BGC0000423 | Scabichelin\_biosynthetic\_gene\_cluster | 27 | 76.1424332344 | 336 | 1.9e-91 |
| AHZ20774.1 | non-ribosomal\_peptide\_synthase | BGC0000369 | Hassallidins\_biosynthetic\_gene\_cluster | 27 | 64.6290801187 | 335 | 3.2e-91 |
| ADL64235.1 | aureusimine\_non-ribosomal\_peptide\_synthetase | BGC0000308 | Aureusimine\_biosynthetic\_gene\_cluster | 25 | 92.5816023739 | 335 | 4.2e-91 |
| ANY57892.1 | PenN | BGC0001372 | Penigequinolone\_biosynthetic\_gene\_cluster | 32 | 41.2462908012 | 335 | 4.2e-91 |
| AEP18655.1 | WAPS2 | BGC0000461 | WAP-8294A2\_(lotilibcin)\_biosynthetic\_gene\_cluster | 28 | 65.5786350148 | 334 | 7.2e-91 |
| CAC48361.1 | peptide\_synthetase | BGC0000311 | Balhimycin\_biosynthetic\_gene\_cluster | 28 | 63.9762611276 | 334 | 9.4e-91 |
| AKP45399.1 | CysK | BGC0001413 | Cystobactamide\_biosynthetic\_gene\_cluster | 24 | 106.587537092 | 333 | 1.6e-90 |
| AAY93354.1 | non-ribosomal\_peptide\_synthetase\_PvdD | BGC0000413 | Pyoverdine\_biosynthetic\_gene\_cluster | 28 | 66.765578635 | 333 | 1.6e-90 |
| CAJ46692.1 | non\_ribosomal\_peptide\_synthase | BGC0000969 | Chondramide\_biosynthetic\_gene\_cluster | 27 | 64.4510385757 | 331 | 6.1e-90 |
| WP\_010369428.1 | phenylalanine\_racemase | BGC0000314 | Bromoalterochromides\_biosynthetic\_gene\_cluster | 27 | 67.1810089021 | 330 | 1e-89 |
| ERG36783.1 | phenylalanine\_racemase | BGC0000299 | Alterochromides\_biosynthetic\_gene\_cluster | 27 | 67.1810089021 | 330 | 1e-89 |
| AHH53507.1 | non-ribosomal\_peptide\_synthetase | BGC0000439 | Taromycin\_biosynthetic\_gene\_cluster | 27 | 64.7477744807 | 330 | 1.4e-89 |
| AGE11899.1 | nonribosomal\_peptide\_synthetase | BGC0000366 | Gobichelin\_biosynthetic\_gene\_cluster | 27 | 64.9851632047 | 329 | 2.3e-89 |
| ALV82384.1 | CDA\_peptide\_synthetase\_II | BGC0001370 | Lipopeptide\_8D1-1\_/\_lipopeptide\_8D1-2\_biosynthetic\_gene\_cluster | 27 | 64.9258160237 | 329 | 3e-89 |
| AJM89735.1 | PmxA | BGC0001192 | Colistin\_biosynthetic\_gene\_cluster | 27 | 65.5192878338 | 328 | 3.9e-89 |
| ABV56588.1 | KtzH | BGC0000378 | Kutznerides\_biosynthetic\_gene\_cluster | 27 | 63.7982195846 | 327 | 8.8e-89 |
| CAD91211.1 | putative\_non-ribosomal\_peptide\_synthetase,\_module\_7 | BGC0000289 | A40926\_biosynthetic\_gene\_cluster | 27 | 64.8664688427 | 326 | 1.5e-88 |
| CAB38518.1 | CDA\_peptide\_synthetase\_I | BGC0000315 | Calcium-dependent\_antibiotic\_biosynthetic\_gene\_cluster | 28 | 64.9258160237 | 326 | 1.5e-88 |
| AEH41793.1 | HrmO | BGC0000374 | Hormaomycin\_biosynthetic\_gene\_cluster | 27 | 64.5103857567 | 325 | 4.4e-88 |
| CAB38517.1 | CDA\_peptide\_synthetase\_II | BGC0000315 | Calcium-dependent\_antibiotic\_biosynthetic\_gene\_cluster | 28 | 65.2225519288 | 324 | 7.4e-88 |
| AIW82277.1 | PuwA | BGC0001125 | Puwainaphycins\_biosynthetic\_gene\_cluster | 26 | 75.9643916914 | 324 | 7.4e-88 |
| CAD91212.1 | putative\_non-ribosomal\_peptide\_synthetase,\_modules\_4-6 | BGC0000289 | A40926\_biosynthetic\_gene\_cluster | 28 | 64.4510385757 | 324 | 9.7e-88 |
| AAF00961.1 | McyB | BGC0001017 | Microcystin\_biosynthetic\_gene\_cluster | 27 | 64.9851632047 | 324 | 9.7e-88 |
| CAM56771.1 |  | BGC0000354 | Friulimicin\_biosynthetic\_gene\_cluster | 27 | 64.8664688427 | 323 | 1.7e-87 |
| CAJ34374.1 | NRPS\_protein | BGC0000445 | Thiocoraline\_biosynthetic\_gene\_cluster | 27 | 64.9851632047 | 322 | 2.8e-87 |
| CAJ96472.1 | Non-ribosomal\_peptide\_synthetase | BGC0000330 | Cupriachelin\_biosynthetic\_gene\_cluster | 26 | 72.5816023739 | 321 | 6.3e-87 |
| AAM80536.1 | StaD | BGC0000290 | A47934\_biosynthetic\_gene\_cluster | 26 | 64.9851632047 | 320 | 1.4e-86 |
| AAT01806.1 | non-ribosomal\_peptide\_synthetase | BGC0000365 | Glycopeptidolipid\_biosynthetic\_gene\_cluster | 26 | 66.350148368 | 319 | 1.8e-86 |
| CAJ77715.1 | Mps1\_protein | BGC0000364 | Glycopeptidolipid\_biosynthetic\_gene\_cluster | 27 | 66.46884273 | 318 | 4.1e-86 |
| ALD82526.1 | non-ribosomal\_peptide\_synthase | BGC0001212 | Nannocystin\_biosynthetic\_gene\_cluster | 27 | 67.9525222552 | 317 | 9.1e-86 |
| AAK81826.1 | peptide\_synthetase | BGC0000326 | Complestatin\_biosynthetic\_gene\_cluster | 26 | 64.9258160237 | 316 | 2e-85 |
| AAC06346.1 | bacitracin\_synthetase\_1 | BGC0000310 | Bacitracin\_biosynthetic\_gene\_cluster | 26 | 65.2818991098 | 315 | 3.5e-85 |
| AHD05618.1 | putative\_non-ribosomal\_peptide\_ligase\_domain\_protein | BGC0001033 | Paenilamicin\_biosynthetic\_gene\_cluster | 25 | 65.9347181009 | 315 | 4.5e-85 |
| EWS95122.1 | hypothetical\_protein | BGC0000306 | Arylomycin\_biosynthetic\_gene\_cluster | 28 | 64.8071216617 | 315 | 4.5e-85 |
| CAJ77696.1 | MPS2\_protein | BGC0000363 | Glycopeptidolipid\_biosynthetic\_gene\_cluster | 26 | 64.6290801187 | 314 | 5.9e-85 |
| AAX31558.1 | peptide\_synthetase\_2 | BGC0000336 | Daptomycin\_biosynthetic\_gene\_cluster | 26 | 64.5697329377 | 313 | 1.7e-84 |
| CAE53353.1 | non-ribosomal\_peptide\_synthetase | BGC0000440 | Teicoplanin\_biosynthetic\_gene\_cluster | 26 | 65.1632047478 | 310 | 1.1e-83 |
| AAU39361.1 | lichenysin\_synthase\_LchAC | BGC0000381 | Lichenysin\_biosynthetic\_gene\_cluster | 26 | 65.6379821958 | 310 | 1.5e-83 |
| CAG15012.1 | peptide\_synthetase,\_module\_7 | BGC0000441 | Teicoplanin\_biosynthetic\_gene\_cluster | 26 | 65.1632047478 | 310 | 1.5e-83 |
| AAD44233.1 | PstA | BGC0000362 | Glycopeptidolipid\_biosynthetic\_gene\_cluster | 26 | 66.1127596439 | 308 | 4.2e-83 |
| AMK48228.1 | nonribosomal\_peptide\_synthetase | BGC0001351 | Cahuitamycins\_biosynthetic\_gene\_cluster | 27 | 64.6290801187 | 308 | 4.2e-83 |
| CAD91220.1 | putative\_non-ribosomal\_peptide\_synthetase,\_modules\_1-2 | BGC0000289 | A40926\_biosynthetic\_gene\_cluster | 29 | 57.8635014837 | 307 | 7.2e-83 |
| AFH75330.1 | nonribosomal\_peptide\_synthetase | BGC0000398 | Orfamide\_biosynthetic\_gene\_cluster | 28 | 57.9228486647 | 307 | 7.2e-83 |
| ABS74181.1 | BmyA | BGC0001090 | Bacillomycin\_biosynthetic\_gene\_cluster | 25 | 72.4629080119 | 307 | 9.4e-83 |
| AAX31559.1 | peptide\_synthetase\_3 | BGC0000336 | Daptomycin\_biosynthetic\_gene\_cluster | 26 | 64.7477744807 | 307 | 9.4e-83 |
| ANI24100.1 | nonribosomal\_peptide\_synthetase | BGC0001235 | Antalid\_biosynthetic\_gene\_cluster | 28 | 58.6943620178 | 307 | 1.2e-82 |
| AAZ55899.1 | amino\_acid\_adenylation | BGC0000359 | Fuscachelin\_biosynthetic\_gene\_cluster | 27 | 64.8664688427 | 306 | 1.6e-82 |
| BAB69698.1 | iturin\_A\_synthetase\_A | BGC0001098 | Iturin\_biosynthetic\_gene\_cluster | 25 | 72.4629080119 | 306 | 2.1e-82 |
| AEG64698.1 | LpmD | BGC0000379 | Laspartomycin\_biosynthetic\_gene\_cluster | 28 | 57.8635014837 | 306 | 2.1e-82 |
| AEI58867.1 | peptide\_synthetase | BGC0000455 | Vancomycin\_biosynthetic\_gene\_cluster | 26 | 65.5786350148 | 305 | 3.6e-82 |
| AJW76710.1 | DsaH | BGC0001196 | Desotamide\_biosynthetic\_gene\_cluster | 25 | 69.9109792285 | 305 | 3.6e-82 |
| AOA33123.1 | nonribosomal\_peptide\_synthetase | BGC0001346 | Bananamides\_biosynthetic\_gene\_cluster | 26 | 65.1632047478 | 305 | 4.7e-82 |
| ADG27359.1 | peptide\_synthetase | BGC0000296 | Actinomycin\_biosynthetic\_gene\_cluster | 26 | 64.8071216617 | 304 | 6.1e-82 |
| CBW45647.1 | PI\_synthetase\_3\_and\_4 | BGC0000952 | Pristinamycin\_biosynthetic\_gene\_cluster | 27 | 65.5192878338 | 304 | 6.1e-82 |
| AGZ15458.1 | putative\_non-ribosomal\_peptide\_synthetase | BGC0001036 | Polyoxypeptin\_biosynthetic\_gene\_cluster | 27 | 65.8753709199 | 304 | 1e-81 |
| AGS77310.1 | NRPS\_module\_7 | BGC0001178 | UK-68,597\_biosynthetic\_gene\_cluster | 26 | 65.1632047478 | 302 | 2.3e-81 |
| AHH53508.1 | non-ribosomal\_peptide\_synthetase | BGC0000439 | Taromycin\_biosynthetic\_gene\_cluster | 27 | 66.1721068249 | 302 | 2.3e-81 |
| CAC48362.1 | peptide\_synthetase | BGC0000311 | Balhimycin\_biosynthetic\_gene\_cluster | 26 | 65.2818991098 | 302 | 3e-81 |
| CAJ77695.1 | MPS1\_protein | BGC0000363 | Glycopeptidolipid\_biosynthetic\_gene\_cluster | 26 | 67.1810089021 | 302 | 3e-81 |
| AHB82071.1 | non\_ribosomal\_peptide\_synthetase | BGC0001231 | Microsclerodermins\_biosynthetic\_gene\_cluster | 33 | 36.7359050445 | 301 | 6.8e-81 |
| CAJ77716.1 | Mps2\_protein | BGC0000364 | Glycopeptidolipid\_biosynthetic\_gene\_cluster | 27 | 64.9851632047 | 301 | 6.8e-81 |
| AAF08795.1 | MycA | BGC0001103 | Mycosubtilin\_biosynthetic\_gene\_cluster | 25 | 72.2255192878 | 299 | 2e-80 |
| AEO14744.1 | NdaB | BGC0000396 | Nodularin\_biosynthetic\_gene\_cluster | 26 | 63.7388724036 | 299 | 3.4e-80 |
| sln7 | nonribosomal\_peptide\_synthetase | BGC0001230 | Salinamides\_biosynthetic\_gene\_cluster | 27 | 65.2225519288 | 298 | 4.4e-80 |
| CAJ14039.1 | peptide\_synthetase\_PhsC | BGC0000406 | Phosphinothricin\_biosynthetic\_gene\_cluster | 27 | 58.1008902077 | 298 | 5.7e-80 |
| AAU34203.1 | mannopeptimycin\_peptide\_synthetase\_MppB | BGC0000388 | Mannopeptimycin\_biosynthetic\_gene\_cluster | 25 | 66.646884273 | 298 | 5.7e-80 |
| CAA11796.1 | PCZA363.5 | BGC0000322 | Chloroeremomycin\_biosynthetic\_gene\_cluster | 25 | 65.4005934718 | 297 | 7.5e-80 |
| BAP27942.1 | nonribosomal\_peptide\_synthetase | BGC0001085 | Teleocidin\_B\_biosynthetic\_gene\_cluster | 25 | 75.8456973294 | 297 | 9.7e-80 |
| AEP18656.1 | WAPS1 | BGC0000461 | WAP-8294A2\_(lotilibcin)\_biosynthetic\_gene\_cluster | 26 | 68.0118694362 | 297 | 1.3e-79 |
| AAZ23075.1 | peptide\_synthetase | BGC0000291 | A54145\_biosynthetic\_gene\_cluster | 25 | 65.7566765579 | 296 | 1.7e-79 |
| AAZ03550.1 | McnA | BGC0000332 | Cyanopeptolin\_biosynthetic\_gene\_cluster | 27 | 66.1127596439 | 294 | 8.3e-79 |
| ALV86868.1 | Tlo22 | BGC0001406 | Telomycin\_biosynthetic\_gene\_cluster | 26 | 64.7477744807 | 294 | 1.1e-78 |
| AIE77060.1 | peptide\_synthetase\_module\_7 | BGC0000418 | Ristocetin\_biosynthetic\_gene\_cluster | 25 | 65.1632047478 | 293 | 1.4e-78 |
| AKC91857.1 | nonribosomal\_peptide\_synthetase | BGC0001414 | Griselimycin\_biosynthetic\_gene\_cluster | 26 | 64.7477744807 | 292 | 3.1e-78 |
| AHB82058.1 | non\_ribosomal\_peptide\_synthetase | BGC0001019 | Microsclerodermins\_biosynthetic\_gene\_cluster | 33 | 39.1691394659 | 291 | 7e-78 |
| CAJ45639.1 | vanchrobactin\_non\_ribosomal\_peptide\_synthetase | BGC0000454 | Vanchrobactin\_biosynthetic\_gene\_cluster | 26 | 65.2225519288 | 290 | 9.1e-78 |
| AAO62588.1 | peptide\_sythetase | BGC0001016 | Microcystin\_biosynthetic\_gene\_cluster | 26 | 64.5103857567 | 290 | 9.1e-78 |
| AAF15891.2 | NosA | BGC0001028 | Nostopeptolide\_biosynthetic\_gene\_cluster | 27 | 65.2818991098 | 289 | 2e-77 |
| ABS74205.1 | FenE | BGC0001095 | Fengycin\_biosynthetic\_gene\_cluster | 25 | 65.5192878338 | 289 | 2e-77 |
| ALK27915.1 | non-ribosomal\_peptide\_synthase | BGC0001233 | Feglymycin\_biosynthetic\_gene\_cluster | 26 | 66.884272997 | 288 | 5.9e-77 |
| CAD29799.1 | microcystin\_synthetase | BGC0001015 | Microcystin\_biosynthetic\_gene\_cluster | 25 | 63.5014836795 | 287 | 1.3e-76 |
| AIG79240.1 | Hypothetical\_protein | BGC0000419 | Ristomycin\_A\_biosynthetic\_gene\_cluster | 25 | 65.0445103858 | 286 | 2.2e-76 |
| AEF33078.1 | dimodular\_nonribosomal\_peptide\_synthetase | BGC0001039 | Pyridomycin\_biosynthetic\_gene\_cluster | 26 | 58.0415430267 | 284 | 6.5e-76 |
| WP\_010369430.1 | Non-ribosomal\_peptide\_synthase | BGC0000314 | Bromoalterochromides\_biosynthetic\_gene\_cluster | 26 | 66.646884273 | 284 | 6.5e-76 |
| CAN89663.1 | putative\_non-ribosomal\_peptide\_synthetase | BGC0001070 | Kirromycin\_biosynthetic\_gene\_cluster | 26 | 66.646884273 | 284 | 8.5e-76 |
| ABI26079.1 | OciC | BGC0000331 | Cyanopeptin\_biosynthetic\_gene\_cluster | 28 | 59.1691394659 | 282 | 2.5e-75 |
| AAF00962.1 | McyC | BGC0001017 | Microcystin\_biosynthetic\_gene\_cluster | 25 | 64.2136498516 | 282 | 4.2e-75 |
| AGS77308.1 | NRPS\_module\_3 | BGC0001178 | UK-68,597\_biosynthetic\_gene\_cluster | 25 | 64.2729970326 | 281 | 5.5e-75 |
| BAH33409.1 | putative\_non-ribosomal\_peptide\_synthetase | BGC0000371 | Heterobactin\_biosynthetic\_gene\_cluster | 26 | 66.824925816 | 281 | 5.5e-75 |
| ACZ55945.1 | non-ribosomal\_peptide\_synthetase | BGC0000302 | Anabaenopeptin\_biosynthetic\_gene\_cluster | 33 | 38.2195845697 | 281 | 7.2e-75 |
| CAG15010.1 | peptide\_synthetase,\_module\_3 | BGC0000441 | Teicoplanin\_biosynthetic\_gene\_cluster | 25 | 64.5103857567 | 281 | 7.2e-75 |
| AIW82282.1 | PuwE | BGC0001125 | Puwainaphycins\_biosynthetic\_gene\_cluster | 27 | 59.4065281899 | 280 | 1.2e-74 |
| BAH22762.1 | nonribosomal\_peptide\_synthetase | BGC0001018 | Micropeptin\_biosynthetic\_gene\_cluster | 27 | 66.528189911 | 280 | 1.2e-74 |
| AAM80538.1 | StaB | BGC0000290 | A47934\_biosynthetic\_gene\_cluster | 25 | 64.6290801187 | 280 | 1.6e-74 |
| AGE25479.1 | putative\_non-ribosomal\_peptide\_synthetase | BGC0001208\_c2 | Poaeamide\_biosynthetic\_gene\_cluster | 25 | 64.5697329377 | 279 | 2.1e-74 |
| CAD91221.1 | putative\_non-ribosomal\_peptide\_synthetase,\_module\_3 | BGC0000289 | A40926\_biosynthetic\_gene\_cluster | 26 | 64.5697329377 | 279 | 2.1e-74 |
| CAE53351.1 | non-ribosomal\_peptide\_synthetase | BGC0000440 | Teicoplanin\_biosynthetic\_gene\_cluster | 25 | 64.5103857567 | 278 | 6.1e-74 |
| AIE77058.1 | peptide\_synthetase\_module\_3 | BGC0000418 | Ristocetin\_biosynthetic\_gene\_cluster | 25 | 64.6884272997 | 278 | 6.1e-74 |
| ABP57749.1 | DepE | BGC0000993 | FK228\_biosynthetic\_gene\_cluster | 26 | 65.4005934718 | 277 | 8e-74 |
| AAO62586.1 | peptide\_sythetase | BGC0001016 | Microcystin\_biosynthetic\_gene\_cluster | 33 | 36.5578635015 | 276 | 2.3e-73 |
| CAE52339.1 | Polyketide\_non-ribosomal\_peptide\_synthase | BGC0001088 | Albicidin\_biosynthetic\_gene\_cluster | 24 | 105.697329377 | 276 | 2.3e-73 |
| CBL93730.1 | NRPS | BGC0000360 | GE81112\_biosynthetic\_gene\_cluster | 25 | 69.9109792285 | 272 | 2.6e-72 |
| AHF21229.1 | TriE | BGC0000449 | Tridecaptin\_biosynthetic\_gene\_cluster | 30 | 49.4955489614 | 271 | 5.7e-72 |
| ABC36785.1 | peptide\_synthetase,\_putative | BGC0000964 | Burkholdac\_biosynthetic\_gene\_cluster | 25 | 64.3916913947 | 270 | 9.8e-72 |
| CAQ34921.1 | nonribosomal\_peptide\_synthetase | BGC0000986 | Dkxanthene\_biosynthetic\_gene\_cluster | 32 | 36.6765578635 | 270 | 1.3e-71 |
| ACM79810.1 | ZmaO | BGC0001059 | Zwittermycin\_A\_biosynthetic\_gene\_cluster | 25 | 63.0860534125 | 269 | 2.8e-71 |
| AFD30953.1 | CrmB | BGC0000966 | Caerulomycin\_A\_biosynthetic\_gene\_cluster | 25 | 66.0534124629 | 268 | 6.3e-71 |
| AJI44167.1 | long-chain-fatty-acid-CoA\_ligase | BGC0001193 | Thiolutin\_biosynthetic\_gene\_cluster | 28 | 51.8100890208 | 268 | 6.3e-71 |
| sln6 | nonribosomal\_peptide\_synthetase | BGC0001230 | Salinamides\_biosynthetic\_gene\_cluster | 25 | 66.943620178 | 267 | 1.4e-70 |
| ALV86867.1 | Tlo21 | BGC0001406 | Telomycin\_biosynthetic\_gene\_cluster | 27 | 55.5489614243 | 266 | 1.8e-70 |
| AGN74886.1 | nonribosomal\_peptide\_synthetase | BGC0000459 | Griseoviridin\_/\_viridogrisein\_biosynthetic\_gene\_cluster | 26 | 67.4183976261 | 266 | 1.8e-70 |
| AHB38497.1 | EpxD | BGC0000346 | Epoxomicin\_biosynthetic\_gene\_cluster | 28 | 58.5756676558 | 265 | 4.1e-70 |
| BAH22765.1 | nonribosomal\_peptide\_synthetase | BGC0001018 | Micropeptin\_biosynthetic\_gene\_cluster | 26 | 58.3382789318 | 265 | 4.1e-70 |
| ctg1\_orf19 |  | BGC0001013 | Meridamycin\_biosynthetic\_gene\_cluster | 27 | 59.881305638 | 264 | 7e-70 |
| WP\_052390334.1 | non-ribosomal\_peptide\_synthetase | BGC0001308\_c2 | Simocyclinone\_D9\_/\_simocyclinone\_D10\_/\_simocyclinone\_D11 | 28 | 49.9109792285 | 264 | 7e-70 |
| ABC87508.1 | NRPS\_for\_pipecolate\_incorporation | BGC0001011 | Meridamycin\_biosynthetic\_gene\_cluster | 27 | 59.881305638 | 264 | 7e-70 |
| AGZ15459.1 | putative\_non-ribosomal\_peptide\_synthetase | BGC0001036 | Polyoxypeptin\_biosynthetic\_gene\_cluster | 27 | 55.7270029674 | 264 | 9.1e-70 |
| ACY06285.1 | non-ribosomal\_peptide\_synthetase | BGC0001042 | Sanglifehrin\_A\_biosynthetic\_gene\_cluster | 27 | 50.6231454006 | 264 | 1.2e-69 |
| ADA69239.2 | trans-AT\_hybrid\_polyketide\_synthase-NRPS | BGC0001071 | Nosperin\_biosynthetic\_gene\_cluster | 26 | 59.1097922849 | 263 | 1.6e-69 |
| CAF05648.1 | TubC\_protein | BGC0001053 | Tubulysin\_biosynthetic\_gene\_cluster | 26 | 59.1691394659 | 263 | 2e-69 |
| ALG65318.1 | Cal18 | BGC0001297 | WS9326\_biosynthetic\_gene\_cluster | 28 | 50.8011869436 | 262 | 3.5e-69 |
| AAY93355.1 | non-ribosomal\_peptide\_synthetase\_PvdJ | BGC0000413 | Pyoverdine\_biosynthetic\_gene\_cluster | 27 | 50.8011869436 | 262 | 3.5e-69 |
| AHD05677.1 | nonribosomal\_peptide\_ligase\_subunit | BGC0000402 | Paenilarvins\_biosynthetic\_gene\_cluster | 29 | 41.0682492582 | 260 | 1.3e-68 |
| AGD80618.1 | non-ribosomal\_peptide\_synthetase | BGC0000394 | Naphthyridinomycin\_biosynthetic\_gene\_cluster | 26 | 59.1097922849 | 260 | 1.3e-68 |
| AFJ14795.1 | PlpF | BGC0000403 | Pelgipeptin\_biosynthetic\_gene\_cluster | 29 | 42.0178041543 | 260 | 1.7e-68 |
| AAX31557.1 | peptide\_synthetase\_1 | BGC0000336 | Daptomycin\_biosynthetic\_gene\_cluster | 27 | 58.6943620178 | 258 | 6.6e-68 |
| CCJ67647.1 | JagC | BGC0001127 | Jagaricin\_biosynthetic\_gene\_cluster | 26 | 63.8575667656 | 257 | 8.6e-68 |
| CAC48369.1 | peptide\_synthetase | BGC0000311 | Balhimycin\_biosynthetic\_gene\_cluster | 33 | 36.0237388724 | 257 | 1.1e-67 |
| AHB82059.1 | non\_ribosomal\_peptide\_synthetase/polyketide\_synthase | BGC0001019 | Microsclerodermins\_biosynthetic\_gene\_cluster | 25 | 58.6350148368 | 257 | 1.1e-67 |
| CCA29203.1 | non-ribosomal\_peptide\_synthetase/polyketide\_synthase | BGC0000955 | Althiomycin\_biosynthetic\_gene\_cluster | 24 | 65.3412462908 | 256 | 1.9e-67 |
| AFR69334.1 | nonribosomal\_peptide\_synthetase\_SpiDE1 | BGC0001045 | Spiruchostatin\_biosynthetic\_gene\_cluster | 25 | 65.1038575668 | 256 | 2.5e-67 |
| ACM68684.1 | AerB | BGC0000298 | Aeruginosin\_biosynthetic\_gene\_cluster | 28 | 49.8516320475 | 255 | 3.3e-67 |
| AJW76711.1 | DsaI | BGC0001196 | Desotamide\_biosynthetic\_gene\_cluster | 27 | 50.9792284866 | 255 | 5.5e-67 |
| AAN65224.1 | peptide\_synthetase-like\_protein | BGC0000832 | Clorobiocin\_biosynthetic\_gene\_cluster | 32 | 36.5578635015 | 254 | 7.2e-67 |
| AGZ03650.1 | SevA | BGC0000426 | Sevadicin\_biosynthetic\_gene\_cluster | 32 | 36.5578635015 | 253 | 1.6e-66 |
| ABF85931.1 | non-ribosomal\_peptide\_synthase/polyketide\_synthase\_Ta1 | BGC0001025 | Myxovirescin\_biosynthetic\_gene\_cluster | 25 | 64.7477744807 | 253 | 2.1e-66 |
| AAY93356.2 | non-ribosomal\_peptide\_synthetase\_PvdI | BGC0000413 | Pyoverdine\_biosynthetic\_gene\_cluster | 31 | 37.3887240356 | 252 | 3.6e-66 |
| AHD05617.1 | putative\_non-ribosomal\_peptide\_ligase\_domain\_protein | BGC0001033 | Paenilamicin\_biosynthetic\_gene\_cluster | 25 | 59.762611276 | 250 | 1e-65 |
| BAJ19066.1 | L-lysine\_activating\_non-ribosomal\_peptide\_synthetase | BGC0000288 | A-503083\_biosynthetic\_gene\_cluster | 24 | 67.4777448071 | 250 | 1e-65 |
| AAN32981.1 | BarG | BGC0000962 | Barbamide\_biosynthetic\_gene\_cluster | 25 | 53.8278931751 | 250 | 1e-65 |
| CBJ90289.1 | peptide\_synthetase | BGC0000416 | Rhabdopeptides\_biosynthetic\_gene\_cluster | 26 | 54.1246290801 | 250 | 1.4e-65 |
| CAG23960.2 | hybrid\_NRPS/PKS | BGC0001089 | Bacillaene\_biosynthetic\_gene\_cluster | 25 | 65.5192878338 | 250 | 1.8e-65 |
| CCJ67645.1 | JagA | BGC0001127 | Jagaricin\_biosynthetic\_gene\_cluster | 27 | 58.6943620178 | 249 | 3e-65 |
| AMK48226.1 | nonribosomal\_peptide\_synthetase | BGC0001351 | Cahuitamycins\_biosynthetic\_gene\_cluster | 33 | 37.0326409496 | 247 | 8.9e-65 |
| CAQ43084.1 | non\_ribosomal\_polypeptide\_synthetase | BGC0000970 | Chondrochloren\_biosynthetic\_gene\_cluster | 26 | 49.8516320475 | 247 | 8.9e-65 |
| EFE73308.1 | non-ribosomal\_peptide\_synthetase | BGC0000431 | Stenothricin\_biosynthetic\_gene\_cluster | 31 | 36.8545994065 | 246 | 2e-64 |
| CAQ71828.1 | non\_ribosomal\_peptide\_synthase,\_antibiotic\_synthesis;\_contains\_3\_condensation\_domains,\_2\_AMP-acid\_ligases\_II\_domains,\_2\_PP-binding,\_Phosphopantetheine\_attachment\_site | BGC0001189 | Taiwachelin\_biosynthetic\_gene\_cluster | 26 | 64.0356083086 | 246 | 2.6e-64 |
| AGE11898.1 | nonribosomal\_peptide\_synthetase | BGC0000366 | Gobichelin\_biosynthetic\_gene\_cluster | 25 | 57.7448071217 | 245 | 3.4e-64 |
| AAC32048.1 | FxbC | BGC0000351 | Exochelin\_biosynthetic\_gene\_cluster | 25 | 60.6528189911 | 245 | 3.4e-64 |
| AJW76709.1 | DsaG | BGC0001196 | Desotamide\_biosynthetic\_gene\_cluster | 30 | 48.9614243323 | 245 | 3.4e-64 |
| CBJ90288.1 | Peptide\_synthetase | BGC0000416 | Rhabdopeptides\_biosynthetic\_gene\_cluster | 25 | 54.2433234421 | 245 | 4.4e-64 |
| CAC11137.1 | NikP1\_protein | BGC0000876 | Nikkomycin\_biosynthetic\_gene\_cluster | 30 | 37.5074183976 | 245 | 5.7e-64 |
| AAT09804.1 | NocA | BGC0000395 | Nocardicin\_A\_biosynthetic\_gene\_cluster | 29 | 57.8635014837 | 244 | 9.8e-64 |
| ABP57748.1 | DepD | BGC0000993 | FK228\_biosynthetic\_gene\_cluster | 30 | 48.1305637982 | 243 | 1.7e-63 |
| CAN89656.1 | putative\_non-ribosomal\_peptide\_synthetase | BGC0001070 | Kirromycin\_biosynthetic\_gene\_cluster | 30 | 49.5548961424 | 243 | 2.2e-63 |
| AJV88377.1 | MfnE | BGC0001214 | Marformycins\_biosynthetic\_gene\_cluster | 29 | 40.6528189911 | 242 | 2.8e-63 |
| ABW00331.1 | amino\_acid\_adenylation\_domain | BGC0000333 | Cyclomarin\_biosynthetic\_gene\_cluster | 32 | 36.0237388724 | 242 | 2.8e-63 |
| ABD65956.1 | nonribosomal\_peptide\_synthetase | BGC0000341 | Enduracidin\_biosynthetic\_gene\_cluster | 26 | 53.471810089 | 241 | 6.3e-63 |
| BAP05596.1 | CalH | BGC0000967 | Calyculin\_biosynthetic\_gene\_cluster | 28 | 37.3887240356 | 241 | 8.3e-63 |
| AIE77076.1 | peptide\_synthetase | BGC0000418 | Ristocetin\_biosynthetic\_gene\_cluster | 30 | 35.6676557864 | 240 | 1.1e-62 |
| AEG64696.1 | LpmB | BGC0000379 | Laspartomycin\_biosynthetic\_gene\_cluster | 27 | 54.9554896142 | 240 | 1.1e-62 |
| AEI70245.1 | nonribosomal\_peptide\_synthetase\_NRPS | BGC0000401 | Paenibactin\_biosynthetic\_gene\_cluster | 27 | 58.1008902077 | 240 | 1.8e-62 |
| EDY47118.1 | N-(5-amino-5-carboxypentanoyl)-L-cysteinyl-D-\_valine\_synthase | BGC0000319 | Cephamycin\_C\_biosynthetic\_gene\_cluster | 27 | 51.3946587537 | 238 | 4.1e-62 |
| AHB82056.1 | non\_ribosomal\_peptide\_synthetase | BGC0001019 | Microsclerodermins\_biosynthetic\_gene\_cluster | 24 | 65.4005934718 | 238 | 5.4e-62 |
| ADG27358.1 | peptide\_synthetase | BGC0000296 | Actinomycin\_biosynthetic\_gene\_cluster | 26 | 53.9465875371 | 238 | 7e-62 |
| AID65222.1 | putative\_aspartate\_racemase | BGC0000335 | Cystomanamides\_biosynthetic\_gene\_cluster | 28 | 48.2492581602 | 238 | 7e-62 |
| CBJ90358.1 | putative\_Peptide\_synthetase | BGC0000465 | Xenortide\_biosynthetic\_gene\_cluster | 25 | 53.590504451 | 238 | 7e-62 |
| CAJ76298.1 | putative\_hybrid\_polyketide-non-ribosomal\_peptide\_synthetase | BGC0000972 | Colibactin\_biosynthetic\_gene\_cluster | 27 | 50.5044510386 | 237 | 1.2e-61 |
| ABV56604.1 | adenylation\_domain\_protein | BGC0000378 | Kutznerides\_biosynthetic\_gene\_cluster | 32 | 35.6676557864 | 237 | 1.6e-61 |
| AHH53506.1 | non-ribosomal\_peptide\_synthetase | BGC0000439 | Taromycin\_biosynthetic\_gene\_cluster | 32 | 36.3798219585 | 237 | 1.6e-61 |
| ABV79988.1 | ApnD | BGC0000301 | Anabaenopeptin\_biosynthetic\_gene\_cluster | 26 | 59.703264095 | 236 | 2e-61 |
| CAJ96470.1 | Non-ribosomal\_peptide\_synthetase | BGC0000330 | Cupriachelin\_biosynthetic\_gene\_cluster | 28 | 48.3679525223 | 236 | 2e-61 |
| ADC79642.1 | TamD | BGC0001052 | Tirandamycin\_biosynthetic\_gene\_cluster | 26 | 57.7448071217 | 235 | 3.5e-61 |
| AHZ34238.1 | CipA | BGC0001389 | Cichopeptin\_biosynthetic\_gene\_cluster | 27 | 41.1275964392 | 235 | 3.5e-61 |
| AAP92496.1 | nonribosomal\_peptide\_synthetase | BGC0000458 | Viomycin\_biosynthetic\_gene\_cluster | 28 | 50.8011869436 | 235 | 5.9e-61 |
| AAN85512.1 | nonribosomal\_peptide\_synthetase | BGC0001101 | Leinamycin\_biosynthetic\_gene\_cluster | 25 | 58.1602373887 | 234 | 1e-60 |
| AHB82069.1 | non\_ribosomal\_peptide\_synthetase | BGC0001231 | Microsclerodermins\_biosynthetic\_gene\_cluster | 31 | 35.1928783383 | 233 | 1.3e-60 |
| ADH01485.1 | putative\_mixed\_polyketide\_synthase/non-ribosomal\_peptide\_synthetase | BGC0000995 | FR901464\_biosynthetic\_gene\_cluster | 26 | 59.0504451039 | 233 | 1.7e-60 |
| AIC32693.1 | FR9DEF | BGC0001113 | Spliceostatin\_/\_FR901464\_biosynthetic\_gene\_cluster | 26 | 59.0504451039 | 233 | 1.7e-60 |
| CAE14171.1 |  | BGC0000383 | Luminmycin\_biosynthetic\_gene\_cluster | 24 | 65.1632047478 | 233 | 2.3e-60 |
| CBD77746.1 | non-ribosomal\_peptide\_synthetase/polyketide\_synthase | BGC0000974 | Crocacin\_biosynthetic\_gene\_cluster | 26 | 58.8724035608 | 233 | 2.3e-60 |
| AHI59110.1 | locillomycin\_synthase\_C | BGC0001005 | Locillomycin\_biosynthetic\_gene\_cluster | 25 | 51.4540059347 | 232 | 3.9e-60 |
| ABM21571.1 | CrpC | BGC0000975 | Cryptophycin\_biosynthetic\_gene\_cluster | 32 | 28.3679525223 | 232 | 5e-60 |
| ABI22132.1 | putative\_non-ribosomal\_peptide\_synthetase | BGC0000422 | Saframycin\_A\_biosynthetic\_gene\_cluster | 26 | 58.6350148368 | 231 | 6.6e-60 |
| CBJ90287.1 | peptide\_synthetase | BGC0000416 | Rhabdopeptides\_biosynthetic\_gene\_cluster | 24 | 53.412462908 | 230 | 1.1e-59 |
| ABD14711.1 | CesA | BGC0000320 | Cereulide\_biosynthetic\_gene\_cluster | 28 | 36.6172106825 | 230 | 1.5e-59 |
| ALV86866.1 | Tlo20 | BGC0001406 | Telomycin\_biosynthetic\_gene\_cluster | 30 | 40.237388724 | 230 | 1.9e-59 |
| CAJ88610.1 | putative\_non-ribosomal\_peptide\_synthetase | BGC0000327 | Congocidine\_biosynthetic\_gene\_cluster | 31 | 37.5667655786 | 229 | 3.3e-59 |
| AID65224.1 | nonribosomal\_peptide\_synthetase | BGC0000335 | Cystomanamides\_biosynthetic\_gene\_cluster | 26 | 49.3768545994 | 228 | 5.6e-59 |
| CAJ18237.2 | non-ribosomal\_peptide\_synthetase\_B | BGC0000354 | Friulimicin\_biosynthetic\_gene\_cluster | 26 | 51.1572700297 | 228 | 5.6e-59 |
| ADY76667.1 | non-ribosomal\_peptide\_synthetase | BGC0000950 | Napsamycin\_biosynthetic\_gene\_cluster | 29 | 38.4569732938 | 227 | 9.5e-59 |
| AHD05627.1 | putative\_non-ribosomal\_peptide\_ligase\_domain\_protein | BGC0001033 | Paenilamicin\_biosynthetic\_gene\_cluster | 23 | 64.2136498516 | 227 | 9.5e-59 |
| ABS75232.1 | DhbF | BGC0001185 | Bacillibactin\_biosynthetic\_gene\_cluster | 26 | 53.234421365 | 226 | 2.1e-58 |
| CDF96614.1 | Long-chain-fatty-acid--CoA\_ligase | BGC0001149 | Brabantamide\_biosynthetic\_gene\_cluster | 25 | 59.4065281899 | 226 | 2.1e-58 |
| AAC68816.1 | FK506\_peptide\_synthetase | BGC0000353 | FK506\_biosynthetic\_gene\_cluster | 25 | 58.7537091988 | 225 | 3.6e-58 |
| ALG65319.1 | Cal17 | BGC0001297 | WS9326\_biosynthetic\_gene\_cluster | 24 | 66.884272997 | 224 | 8e-58 |
| BAO84866.1 | putative\_non-ribosomal\_peptide\_synthetase | BGC0000414 | Quinocarcin\_biosynthetic\_gene\_cluster | 24 | 58.6350148368 | 224 | 1e-57 |
| AHB38515.1 | EpnG | BGC0000345 | Eponemycin\_biosynthetic\_gene\_cluster | 30 | 38.4569732938 | 224 | 1e-57 |
| ABC35522.1 | thiotemplate\_mechanism\_natural\_product\_synthetase | BGC0000186 | Thailandamide\_biosynthetic\_gene\_cluster | 30 | 36.7952522255 | 224 | 1e-57 |
| AIG79242.1 | Hypothetical\_protein | BGC0000419 | Ristomycin\_A\_biosynthetic\_gene\_cluster | 24 | 52.9376854599 | 223 | 1.4e-57 |
| AHB82072.1 | non\_ribosomal\_peptide\_synthetase/polyketide\_synthase | BGC0001231 | Microsclerodermins\_biosynthetic\_gene\_cluster | 25 | 48.3679525223 | 223 | 1.8e-57 |
| AAZ23076.1 | peptide\_synthetase | BGC0000291 | A54145\_biosynthetic\_gene\_cluster | 30 | 36.7952522255 | 223 | 1.8e-57 |
| AIG79224.1 | Hypothetical\_protein | BGC0000419 | Ristomycin\_A\_biosynthetic\_gene\_cluster | 30 | 36.0237388724 | 223 | 1.8e-57 |
| ABV56585.1 | KtzE | BGC0000378 | Kutznerides\_biosynthetic\_gene\_cluster | 31 | 36.3204747774 | 222 | 3.1e-57 |
| AAT09805.1 | NocB | BGC0000395 | Nocardicin\_A\_biosynthetic\_gene\_cluster | 29 | 38.0415430267 | 222 | 5.2e-57 |
| CAD70195.1 | non-ribosomal\_peptide\_synthetase | BGC0001047 | Syringolin\_A\_biosynthetic\_gene\_cluster | 31 | 36.0830860534 | 222 | 5.2e-57 |
| CCJ67646.1 | JagB | BGC0001127 | Jagaricin\_biosynthetic\_gene\_cluster | 31 | 36.3798219585 | 222 | 5.2e-57 |
| ABI22133.1 | putative\_non-ribosomal\_peptide\_synthetase | BGC0000422 | Saframycin\_A\_biosynthetic\_gene\_cluster | 25 | 59.1691394659 | 221 | 6.8e-57 |
| CCC55922.1 | putative\_non-ribosomal\_peptide\_synthetase | BGC0000973 | Collismycin\_A\_biosynthetic\_gene\_cluster | 25 | 59.6439169139 | 221 | 6.8e-57 |
| EFL06865.1 | hypothetical\_protein | BGC0000300 | Amychelin\_biosynthetic\_gene\_cluster | 31 | 36.0237388724 | 219 | 2.6e-56 |
| ABA23460.1 | Amino\_acid\_adenylation | BGC0000427 | Shinorine\_biosynthetic\_gene\_cluster | 28 | 36.6172106825 | 219 | 2.6e-56 |
| ADY76664.1 | non-ribosomal\_peptide\_synthetase | BGC0000950 | Napsamycin\_biosynthetic\_gene\_cluster | 31 | 37.2106824926 | 219 | 2.6e-56 |
| AEA30272.1 | peptide\_synthetase | BGC0000429 | Skyllamycin\_biosynthetic\_gene\_cluster | 30 | 37.6854599407 | 219 | 3.4e-56 |
| AAG02349.1 | peptide\_synthetase\_NRPS11-10 | BGC0000963 | Bleomycin\_biosynthetic\_gene\_cluster | 24 | 73.175074184 | 219 | 3.4e-56 |
| CAE15637.1 |  | BGC0001128 | Luminmide\_biosynthetic\_gene\_cluster | 29 | 37.9228486647 | 218 | 4.4e-56 |
| ALG65317.1 | Cal19 | BGC0001297 | WS9326\_biosynthetic\_gene\_cluster | 31 | 36.9139465875 | 218 | 4.4e-56 |
| BAP05591.1 | CalC | BGC0000967 | Calyculin\_biosynthetic\_gene\_cluster | 29 | 36.9139465875 | 218 | 7.5e-56 |
| AGN74885.1 | nonribosomal\_peptide\_synthetase | BGC0000459 | Griseoviridin\_/\_viridogrisein\_biosynthetic\_gene\_cluster | 25 | 54.8367952522 | 217 | 9.8e-56 |
| BAP05590.1 | CalB | BGC0000967 | Calyculin\_biosynthetic\_gene\_cluster | 29 | 36.7359050445 | 217 | 9.8e-56 |
| ABI22131.1 | putative\_non-ribosomal\_peptide\_synthetase | BGC0000422 | Saframycin\_A\_biosynthetic\_gene\_cluster | 25 | 65.6973293769 | 217 | 1.3e-55 |
| CBZ42143.1 | putative\_non-ribosomal\_peptide\_synthetase | BGC0001117 | Himastatin\_biosynthetic\_gene\_cluster | 30 | 36.5578635015 | 217 | 1.7e-55 |
| ALG65314.1 | Cal22 | BGC0001297 | WS9326\_biosynthetic\_gene\_cluster | 27 | 42.3145400593 | 216 | 2.2e-55 |
| WP\_010369425.1 | ornithine\_racemase | BGC0000314 | Bromoalterochromides\_biosynthetic\_gene\_cluster | 29 | 38.5756676558 | 215 | 3.7e-55 |
| ERG36782.1 | Ornithine\_racemase | BGC0000299 | Alterochromides\_biosynthetic\_gene\_cluster | 29 | 38.5756676558 | 215 | 3.7e-55 |
| ADH04680.1 | hybrid\_polyketide\_synthase/non-ribosomal\_peptide\_synthetase | BGC0001344 | Tubulysin\_biosynthetic\_gene\_cluster | 23 | 74.5994065282 | 215 | 3.7e-55 |
| BAE98155.1 | putative\_non-ribosomal\_peptide\_synthetase | BGC0000339 | Echinomycin\_biosynthetic\_gene\_cluster | 27 | 50.5637982196 | 215 | 4.9e-55 |
| AGN11881.1 | TstDEF | BGC0001114 | Thailanstatin\_biosynthetic\_gene\_cluster | 25 | 55.4896142433 | 215 | 4.9e-55 |
| WP\_051700111.1 | non-ribosomal\_peptide\_synthetase | BGC0001368 | Tambromycin\_biosynthetic\_gene\_cluster | 30 | 37.8041543027 | 215 | 4.9e-55 |
| AHH25592.1 | NRPS | BGC0000957 | Ansatrienin\_(mycotrienin)\_biosynthetic\_gene\_cluster | 32 | 36.2017804154 | 215 | 4.9e-55 |
| CCA29202.1 | non-ribosomal\_peptide\_synthetase | BGC0000955 | Althiomycin\_biosynthetic\_gene\_cluster | 29 | 37.5074183976 | 215 | 6.4e-55 |
| AKA59447.1 | non-ribosomal\_peptide\_synthetase | BGC0001203 | Clarexpoxcin\_biosynthetic\_gene\_cluster | 28 | 40.8902077151 | 215 | 6.4e-55 |
| BAF50711.1 | non\_ribosomal\_peptide\_synthetase\_for\_virginiamycin\_S | BGC0001116 | Virginiamycin\_biosynthetic\_gene\_cluster | 28 | 41.5430267062 | 214 | 8.3e-55 |
| AID65225.1 | nonribosomal\_peptide\_synthetase | BGC0000335 | Cystomanamides\_biosynthetic\_gene\_cluster | 26 | 50.2670623145 | 214 | 8.3e-55 |
| CBL93718.1 | NRPS\_didomain\_PCP-C | BGC0000360 | GE81112\_biosynthetic\_gene\_cluster | 25 | 58.4569732938 | 213 | 2.4e-54 |
| BAC67534.2 | arthrofactin\_synthetase\_A | BGC0000305 | Arthrofactin\_biosynthetic\_gene\_cluster | 30 | 38.1602373887 | 212 | 4.1e-54 |
| CAJ14037.1 | peptide\_synthetase\_PhsB | BGC0000406 | Phosphinothricin\_biosynthetic\_gene\_cluster | 25 | 50.6231454006 | 212 | 5.4e-54 |
| ABY83163.1 | Azi25 | BGC0000960 | Azinomycin\_B\_biosynthetic\_gene\_cluster | 24 | 65.8160237389 | 211 | 7e-54 |
| ADI59531.1 | CorI | BGC0001091 | Corallopyronin\_biosynthetic\_gene\_cluster | 29 | 37.0326409496 | 210 | 1.2e-53 |
| WP\_051700105.1 | non-ribosomal\_peptide\_synthetase | BGC0001368 | Tambromycin\_biosynthetic\_gene\_cluster | 29 | 37.0919881306 | 210 | 1.2e-53 |
| CBL93723.1 | NRPS\_didomain\_A-PCP | BGC0000360 | GE81112\_biosynthetic\_gene\_cluster | 30 | 36.8545994065 | 210 | 1.2e-53 |
| CAJ76290.1 | putative\_non-ribosomal\_peptide\_synthase | BGC0000972 | Colibactin\_biosynthetic\_gene\_cluster | 26 | 66.884272997 | 210 | 2e-53 |
| ALJ49909.1 | TlmI | BGC0001237 | Thiolactomycin\_biosynthetic\_gene\_cluster | 29 | 38.3976261128 | 209 | 2.7e-53 |
| CAE52334.1 | non-ribosomal\_peptide\_synthase | BGC0001088 | Albicidin\_biosynthetic\_gene\_cluster | 27 | 47.7151335312 | 208 | 4.6e-53 |
| CCA89328.1 | mixed\_trans-AT\_type\_I\_polyketide\_synthase/nonribosomal\_peptide\_synthetase | BGC0001111 | Rhizopodins\_biosynthetic\_gene\_cluster | 28 | 36.6172106825 | 208 | 7.8e-53 |
| AAF63833.1 | PstD | BGC0000362 | Glycopeptidolipid\_biosynthetic\_gene\_cluster | 27 | 51.3946587537 | 207 | 1e-52 |
| ALD82525.1 | non-ribosomal\_peptide\_synthase | BGC0001212 | Nannocystin\_biosynthetic\_gene\_cluster | 33 | 25.7566765579 | 207 | 1e-52 |
| AAY91419.3 | non-ribosomal\_peptide\_synthetase\_OfaA | BGC0000399 | Orfamide\_biosynthetic\_gene\_cluster | 25 | 46.528189911 | 206 | 2.3e-52 |
| AAY37650.1 | Amino\_acid\_adenylation | BGC0000437 | Syringomycin\_biosynthetic\_gene\_cluster | 28 | 37.0919881306 | 206 | 3e-52 |
| AKA59436.1 | non-ribosomal\_peptide\_synthetase | BGC0001202 | Landepoxcin\_biosynthetic\_gene\_cluster | 26 | 50.5637982196 | 205 | 3.9e-52 |
| ADN26248.1 | peptide\_synthetase | BGC0000951 | Pacidamycin\_biosynthetic\_gene\_cluster | 29 | 37.2106824926 | 205 | 3.9e-52 |
| AAC32047.1 | FxbB | BGC0000351 | Exochelin\_biosynthetic\_gene\_cluster | 29 | 38.9910979228 | 205 | 5e-52 |
| AFU82614.1 | mixed\_NRPS\_PKS | BGC0000998 | Guadinomine\_biosynthetic\_gene\_cluster | 30 | 35.9643916914 | 204 | 1.1e-51 |
| AAN85501.1 | nonribosomal\_peptide\_synthetase | BGC0001101 | Leinamycin\_biosynthetic\_gene\_cluster | 24 | 58.0415430267 | 204 | 1.1e-51 |
| CAN89638.1 | putative\_non-ribosomal\_peptide\_synthetase | BGC0001070 | Kirromycin\_biosynthetic\_gene\_cluster | 30 | 36.6765578635 | 203 | 1.5e-51 |
| CAL80821.1 | sylD-like\_NRPS/PKS | BGC0000997 | Glidobactin\_biosynthetic\_gene\_cluster | 29 | 37.3887240356 | 203 | 1.9e-51 |
| AAY32966.1 | DszC | BGC0001093 | Disorazole\_biosynthetic\_gene\_cluster | 28 | 37.0919881306 | 202 | 3.3e-51 |
| ABP53497.1 | amino\_acid\_adenylation\_domain | BGC0001041 | Salinosporamide\_biosynthetic\_gene\_cluster | 28 | 36.9139465875 | 202 | 4.3e-51 |
| ABP73646.1 | SalB | BGC0000145 | Salinosporamide\_A\_biosynthetic\_gene\_cluster | 28 | 36.9139465875 | 202 | 4.3e-51 |
| CBA63680.1 | nonribosomal\_peptide\_synthetase\_NRPS | BGC0000368 | Griseobactin\_biosynthetic\_gene\_cluster | 26 | 41.1275964392 | 201 | 5.6e-51 |
| CAB53322.1 | putative\_peptide\_synthetase | BGC0000325 | Coelichelin\_biosynthetic\_gene\_cluster | 30 | 37.3293768546 | 201 | 9.5e-51 |
| AAW49318.1 | thaxtomin\_synthetase\_A | BGC0000444 | Thaxtomin\_biosynthetic\_gene\_cluster | 33 | 24.8071216617 | 200 | 1.2e-50 |
| AAT70101.1 | CurF | BGC0001165 | Curacin\_biosynthetic\_gene\_cluster | 26 | 42.9673590504 | 200 | 1.6e-50 |
| AEE88284.1 | CurF | BGC0000976 | Curacin\_biosynthetic\_gene\_cluster | 26 | 42.9673590504 | 200 | 1.6e-50 |
| sln9 | nonribosomal\_peptide\_synthetase | BGC0001230 | Salinamides\_biosynthetic\_gene\_cluster | 25 | 53.293768546 | 200 | 1.6e-50 |
| CAQ71827.1 | non\_ribosomal\_peptide\_synthase,\_antibiotic\_synthesis;\_contains\_1\_condensation\_domain,\_1\_AMP-acid\_ligases\_II\_domain | BGC0001189 | Taiwachelin\_biosynthetic\_gene\_cluster | 26 | 41.9584569733 | 200 | 1.6e-50 |
| AAY42397.1 | HctE | BGC0001000 | Hectochlorin\_biosynthetic\_gene\_cluster | 22 | 70.5637982196 | 199 | 2.8e-50 |
| AFK57219.1 | DidH | BGC0000985 | Didemnin\_biosynthetic\_gene\_cluster | 24 | 63.2640949555 | 199 | 2.8e-50 |
| ABL74945.1 | TlmIV | BGC0001048 | Tallysomycin\_biosynthetic\_gene\_cluster | 28 | 40.5341246291 | 199 | 3.6e-50 |
| ABM21572.1 | CrpD | BGC0000975 | Cryptophycin\_biosynthetic\_gene\_cluster | 25 | 46.350148368 | 198 | 4.7e-50 |
| ABR67749.1 | CmnF | BGC0000316 | Capreomycin\_biosynthetic\_gene\_cluster | 28 | 41.6617210682 | 198 | 6.2e-50 |
| AAG02364.1 | peptide\_synthetase\_NRPS2-1 | BGC0000963 | Bleomycin\_biosynthetic\_gene\_cluster | 28 | 37.9821958457 | 198 | 8e-50 |
| AGD80616.1 | amino\_acid\_adenylation\_domain-containing\_protein | BGC0000394 | Naphthyridinomycin\_biosynthetic\_gene\_cluster | 30 | 31.0979228487 | 198 | 8e-50 |
| EFL06864.1 | non-ribosomal\_peptide\_synthetase | BGC0000300 | Amychelin\_biosynthetic\_gene\_cluster | 24 | 50.5637982196 | 197 | 1.1e-49 |
| CCP45167.1 | Peptide\_synthetase\_MbtF\_(peptide\_synthase) | BGC0001021 | Mycobactin\_biosynthetic\_gene\_cluster | 29 | 37.0919881306 | 196 | 1.8e-49 |
| CAA60461.1 | pipecolate\_incorporating\_enzyme | BGC0001040 | Rapamycin\_biosynthetic\_gene\_cluster | 25 | 66.1127596439 | 196 | 1.8e-49 |
| BAI63288.1 | putative\_non-ribosomal\_peptide\_synthetase | BGC0000434 | SW-163\_biosynthetic\_gene\_cluster | 29 | 35.7270029674 | 196 | 1.8e-49 |
| AAN85506.1 | nonribosomal\_peptide\_synthetase | BGC0001101 | Leinamycin\_biosynthetic\_gene\_cluster | 27 | 37.8041543027 | 195 | 4e-49 |
| ABD65960.1 | nonribosomal\_peptide\_synthetase | BGC0000341 | Enduracidin\_biosynthetic\_gene\_cluster | 28 | 38.9910979228 | 195 | 6.8e-49 |
| CDG12864.1 | non-ribosomal\_peptide\_synthetase | BGC0001415 | Althiomycin\_biosynthetic\_gene\_cluster | 27 | 41.7804154303 | 194 | 8.9e-49 |
| CAF05649.1 | TubD\_protein | BGC0001053 | Tubulysin\_biosynthetic\_gene\_cluster | 22 | 66.1721068249 | 193 | 2e-48 |
| CCC55927.1 | hypothetical\_protein | BGC0000973 | Collismycin\_A\_biosynthetic\_gene\_cluster | 33 | 27.0623145401 | 190 | 1.3e-47 |
| CAD29798.1 | peptide\_synthetase | BGC0001015 | Microcystin\_biosynthetic\_gene\_cluster | 23 | 55.3709198813 | 190 | 2.2e-47 |
| AMK48225.1 | nonribosomal\_peptide\_synthetase | BGC0001351 | Cahuitamycins\_biosynthetic\_gene\_cluster | 28 | 37.3293768546 | 189 | 3.7e-47 |
| CCA89326.1 | mixed\_trans-AT\_type\_I\_polyketide\_synthase/nonribosomal\_peptide\_synthetase | BGC0001111 | Rhizopodins\_biosynthetic\_gene\_cluster | 32 | 31.7507418398 | 187 | 1.1e-46 |
| CAE52342.1 | non-ribosomal\_peptide\_synthase | BGC0001088 | Albicidin\_biosynthetic\_gene\_cluster | 32 | 25.7566765579 | 186 | 1.9e-46 |
| WP\_051700122.1 | non-ribosomal\_peptide\_synthetase | BGC0001368 | Tambromycin\_biosynthetic\_gene\_cluster | 27 | 40.059347181 | 185 | 5.4e-46 |
| EFG10344.1 | Non-ribosomal\_peptide\_synthetase | BGC0000373 | Holomycin\_biosynthetic\_gene\_cluster | 24 | 49.9109792285 | 185 | 7.1e-46 |
| CCC55921.1 | non-ribosomal\_peptide\_synthetase/polyketide\_synthase\_hybrid\_protein | BGC0000973 | Collismycin\_A\_biosynthetic\_gene\_cluster | 28 | 36.7952522255 | 184 | 9.2e-46 |
| AED90004.1 | non-ribosomal\_peptide\_synthetase\_ThaC1 | BGC0000443 | Thanamycin\_biosynthetic\_gene\_cluster | 27 | 37.2106824926 | 184 | 1.2e-45 |
| WP\_028678148.1 | non-ribosomal\_peptide\_synthetase | BGC0001228 | Retimycin\_biosynthetic\_gene\_cluster | 25 | 43.5608308605 | 184 | 1.2e-45 |
| BAP16684.1 | nonribosomal\_peptide\_synthetase | BGC0000376 | JBIR-34,\_JBIR-35\_biosynthetic\_gene\_cluster | 27 | 36.1424332344 | 184 | 1.2e-45 |
| AAG02358.1 | peptide\_synthetase\_NRPS6 | BGC0000963 | Bleomycin\_biosynthetic\_gene\_cluster | 25 | 57.6261127596 | 183 | 1.6e-45 |
| ABJ97436.1 | MerP | BGC0001012 | Meridamycin\_biosynthetic\_gene\_cluster | 31 | 29.1988130564 | 183 | 1.6e-45 |
| ABA59548.1 | Vlm2 | BGC0000453 | Valinomycin\_biosynthetic\_gene\_cluster | 29 | 35.7863501484 | 183 | 1.6e-45 |
| ACG60772.1 | NRPS(C/A/PCP/Cy/A/PCP/Cy) | BGC0001058 | Zorbamycin\_biosynthetic\_gene\_cluster | 25 | 59.2284866469 | 183 | 2.1e-45 |
| AFK57214.1 | DidC | BGC0000985 | Didemnin\_biosynthetic\_gene\_cluster | 24 | 64.4510385757 | 181 | 6e-45 |
| AKC91849.1 | nonribosomal\_peptide\_synthetase | BGC0001414 | Griselimycin\_biosynthetic\_gene\_cluster | 31 | 25.9347181009 | 181 | 6e-45 |
| CAL69597.1 | nonribosomal\_peptide\_synthase | BGC0001049 | Tenellin\_biosynthetic\_gene\_cluster | 23 | 63.6795252226 | 181 | 6e-45 |
| ABC34483.1 | nonribosomal\_peptide\_synthetase,\_putative | BGC0000961 | Bactobolin\_biosynthetic\_gene\_cluster | 27 | 36.4985163205 | 181 | 1e-44 |
| ADN26251.1 | ATP-dependent\_adenylase | BGC0000951 | Pacidamycin\_biosynthetic\_gene\_cluster | 31 | 30.3264094955 | 180 | 1.3e-44 |
| ABP55169.1 | amino\_acid\_adenylation\_domain | BGC0000150 | Sporolide\_biosynthetic\_gene\_cluster | 27 | 36.5578635015 | 180 | 1.3e-44 |
| CAJ96471.1 | Non-ribosomal\_peptide\_synthetase | BGC0000330 | Cupriachelin\_biosynthetic\_gene\_cluster | 26 | 44.4510385757 | 180 | 1.7e-44 |
| ALV82447.1 | putative\_non-ribosomal\_peptide\_synthetase | BGC0001382 | Svaricin\_biosynthetic\_gene\_cluster | 28 | 35.4896142433 | 178 | 5.1e-44 |
| AAW03326.1 | CtaC | BGC0000982 | Cystothiazole\_A\_biosynthetic\_gene\_cluster | 23 | 58.0415430267 | 178 | 6.6e-44 |
| AKP45395.1 | CysG | BGC0001413 | Cystobactamide\_biosynthetic\_gene\_cluster | 26 | 39.2284866469 | 177 | 1.1e-43 |
| KPN90374.1 | NunB1 | BGC0001416 | Nunapeptin\_/\_nunamycin\_biosynthetic\_gene\_cluster | 31 | 25.9940652819 | 177 | 1.1e-43 |
| EOY45602.1 | non-ribosomal\_peptide\_synthetase | BGC0001168 | Livipeptin\_biosynthetic\_gene\_cluster | 28 | 35.1928783383 | 177 | 1.5e-43 |
| AEC14349.1 | nonribosomal\_peptide\_synthetase | BGC0000377 | Koranimine\_biosynthetic\_gene\_cluster | 27 | 35.4896142433 | 176 | 2.5e-43 |
| ABS90470.1 | NRPS/PKS | BGC0001106 | Oxazolomycin\_biosynthetic\_gene\_cluster | 28 | 34.4807121662 | 175 | 4.3e-43 |
| CAQ18839.1 | hybrid\_polyketide\_synthase/nonribosomal\_polypetide\_synthetase | BGC0000954 | Ajudazol\_biosynthetic\_gene\_cluster | 33 | 24.6884272997 | 175 | 5.6e-43 |
| BAP05589.1 | CalA | BGC0000967 | Calyculin\_biosynthetic\_gene\_cluster | 31 | 26.0534124629 | 173 | 2.8e-42 |
| CUI25672.1 | Thiolactomycin\_Hybrid\_PKS-NRPS\_system | BGC0001353 | Thiolactomycin\_biosynthetic\_gene\_cluster | 25 | 37.2700296736 | 173 | 2.8e-42 |
| EAU36744.1 | predicted\_protein | BGC0000292 | Acetylaranotin\_biosynthetic\_gene\_cluster | 27 | 36.2017804154 | 172 | 3.6e-42 |
| ACN39728.1 | SibE | BGC0000428 | Sibiromycin\_biosynthetic\_gene\_cluster | 27 | 36.2611275964 | 171 | 8.1e-42 |
| AGC09519.1 | AMP-dependent\_synthetase/ligase | BGC0001183 | Lobophorin\_biosynthetic\_gene\_cluster | 25 | 39.821958457 | 171 | 1.1e-41 |
| AGZ15476.1 | putative\_non\_ribosomal\_peptide\_synthetase | BGC0001036 | Polyoxypeptin\_biosynthetic\_gene\_cluster | 23 | 55.1928783383 | 171 | 1.1e-41 |
| AAN85493.1 | nonribosomal\_peptide\_synthetase | BGC0001101 | Leinamycin\_biosynthetic\_gene\_cluster | 22 | 54.4213649852 | 170 | 1.8e-41 |
| AGN74898.1 | nonribosomal\_peptide\_synthetase | BGC0000459 | Griseoviridin\_/\_viridogrisein\_biosynthetic\_gene\_cluster | 30 | 31.5133531157 | 169 | 3.1e-41 |
| ABX60161.1 | mixed\_NRPS/PKS | BGC0000978 | Cylindrospermopsin\_biosynthetic\_gene\_cluster | 31 | 29.1394658754 | 169 | 3.1e-41 |
| ADF88262.1 | mixed\_nonribosomal\_peptide\_synthetase/\_polyketide\_synthase | BGC0000979 | Cylindrospermopsin\_biosynthetic\_gene\_cluster | 31 | 29.1394658754 | 168 | 5.2e-41 |
| ADF88265.1 | mixed\_nonribosomal\_peptide\_synthetase/\_polyketide\_synthase | BGC0000980 | Cylindrospermopsin\_biosynthetic\_gene\_cluster | 31 | 29.1394658754 | 168 | 5.2e-41 |
| ADF88279.1 | mixed\_NRPS/PKS | BGC0000981 | Cylindrospermopsin\_biosynthetic\_gene\_cluster | 31 | 29.1394658754 | 168 | 5.2e-41 |
| ABR67758.1 | CmnO | BGC0000316 | Capreomycin\_biosynthetic\_gene\_cluster | 27 | 37.5074183976 | 167 | 1.2e-40 |
| AAF26925.1 | nonribosomal\_peptide\_synthetase | BGC0000988 | Epothilone\_biosynthetic\_gene\_cluster | 24 | 56.4391691395 | 167 | 1.5e-40 |
| BAE98156.1 | putative\_non-ribosomal\_peptide\_synthetase | BGC0000339 | Echinomycin\_biosynthetic\_gene\_cluster | 31 | 25.5192878338 | 166 | 2.6e-40 |
| AAY42398.1 | HctF | BGC0001000 | Hectochlorin\_biosynthetic\_gene\_cluster | 26 | 33.9465875371 | 165 | 5.8e-40 |
| CAC17499.1 | putative\_non-ribosomal\_peptide\_synthase | BGC0000324 | Coelibactin\_biosynthetic\_gene\_cluster | 25 | 36.6765578635 | 164 | 1.3e-39 |
| CBW45749.1 | hybrid\_PKS/NRPS\_(PKSI) | BGC0000952\_c3 | Pristinamycin\_biosynthetic\_gene\_cluster | 24 | 49.7329376855 | 163 | 1.7e-39 |
| CAD15508.1 | probable\_polyketide\_synthase\_protein | BGC0001014 | Micacocidin\_biosynthetic\_gene\_cluster | 28 | 29.2581602374 | 163 | 1.7e-39 |
| ACB46193.1 | nonribosomal\_peptide\_synthetase | BGC0000989 | Epothilone\_biosynthetic\_gene\_cluster | 25 | 43.3234421365 | 162 | 3.7e-39 |
| ADB12489.1 | EpoB | BGC0000990 | Epothilone\_biosynthetic\_gene\_cluster | 25 | 41.1869436202 | 160 | 1.9e-38 |
| AAF62881.1 | EpoB | BGC0000991 | Epothilone\_biosynthetic\_gene\_cluster | 25 | 41.8991097923 | 160 | 2.4e-38 |
| ADQ74618.1 | amino\_acid\_adenylation\_protein | BGC0000921 | Promysalin\_biosynthetic\_gene\_cluster | 26 | 37.5074183976 | 159 | 4.1e-38 |
| BAP16689.1 | nonribosomal\_peptide\_synthetase | BGC0000376 | JBIR-34,\_JBIR-35\_biosynthetic\_gene\_cluster | 24 | 41.1275964392 | 158 | 5.4e-38 |
| AAP92497.1 | nonribosomal\_peptide\_synthetase | BGC0000458 | Viomycin\_biosynthetic\_gene\_cluster | 26 | 38.8724035608 | 158 | 7.1e-38 |
| AJI44176.1 | nonribosomal\_peptide\_synthetase | BGC0001193 | Thiolutin\_biosynthetic\_gene\_cluster | 24 | 37.4480712166 | 158 | 7.1e-38 |
| BAH04161.1 | putative\_non-ribosomal\_peptide\_synthetase | BGC0000450 | Triostin\_A\_biosynthetic\_gene\_cluster | 29 | 25.4005934718 | 158 | 9.2e-38 |
| ADZ45324.1 | non-ribosomal\_peptide\_synthetase | BGC0001020 | Muraymycin\_biosynthetic\_gene\_cluster | 23 | 59.1691394659 | 156 | 2.1e-37 |
| BAI63289.1 | putative\_non-ribosomal\_peptide\_synthetase | BGC0000434 | SW-163\_biosynthetic\_gene\_cluster | 27 | 25.5192878338 | 156 | 2.1e-37 |
| AFD30954.1 | CrmA | BGC0000966 | Caerulomycin\_A\_biosynthetic\_gene\_cluster | 26 | 36.3204747774 | 156 | 2.7e-37 |
| AET98905.1 | putative\_non-ribosomal\_peptide\_synthetase | BGC0000415 | Quinomycin\_biosynthetic\_gene\_cluster | 29 | 25.4005934718 | 156 | 3.5e-37 |
| AAZ55900.1 | non-ribosomal\_peptide\_synthase:Amino\_acid\_adenylation | BGC0000359 | Fuscachelin\_biosynthetic\_gene\_cluster | 30 | 25.8753709199 | 155 | 7.8e-37 |
| AAG05690.1 | AmbE | BGC0000287 | 2-amino-4-methoxy-trans-3-butenoic\_acid\_biosynthetic\_gene\_cluster | 27 | 30.5637982196 | 154 | 1.3e-36 |
| CAQ18831.1 | nonribosomal\_polypetide\_synthetase | BGC0000954 | Ajudazol\_biosynthetic\_gene\_cluster | 23 | 51.8100890208 | 151 | 6.6e-36 |
| AGC65517.1 | TtbB | BGC0001050 | Thalassospiramide\_biosynthetic\_gene\_cluster | 28 | 30.7418397626 | 151 | 6.6e-36 |
| AAD24881.1 | putative\_acyl-CoA\_synthetase | BGC0000127 | Pyoluteorin\_biosynthetic\_gene\_cluster | 28 | 32.3442136499 | 150 | 1.5e-35 |
| EAW09117.1 | hybrid\_NRPS/PKS\_enzyme,\_putative | BGC0000983 | Cytochalasin\_biosynthetic\_gene\_cluster | 23 | 64.8664688427 | 149 | 2.5e-35 |
| ABX71111.1 | Lct28 | BGC0000238 | Lactonamycin\_biosynthetic\_gene\_cluster | 27 | 32.3442136499 | 149 | 2.5e-35 |
| CAJ76289.1 | putative\_hybrid\_non-ribosomal\_peptide-polyketide\_synthetase | BGC0000972 | Colibactin\_biosynthetic\_gene\_cluster | 27 | 27.5964391691 | 149 | 4.3e-35 |
| CAC17500.1 | putative\_non-ribosomal\_peptide\_synthase | BGC0000324 | Coelibactin\_biosynthetic\_gene\_cluster | 23 | 52.3442136499 | 149 | 4.3e-35 |
| ADQ55476.1 | non-ribosomal\_peptide\_synthase\_dimodule | BGC0000350 | ET-743\_biosynthetic\_gene\_cluster | 24 | 37.5074183976 | 149 | 4.3e-35 |
| AFK57221.1 | DidJ | BGC0000985 | Didemnin\_biosynthetic\_gene\_cluster | 31 | 24.6884272997 | 146 | 2.8e-34 |
| CAA46313.1 | peptide\_synthetase\_PhsA | BGC0000406 | Phosphinothricin\_biosynthetic\_gene\_cluster | 26 | 37.0326409496 | 144 | 1.4e-33 |
| ACO94492.1 | NRPS\_adenylation\_domain\_protein | BGC0000097 | ML-449\_biosynthetic\_gene\_cluster | 28 | 25.9940652819 | 143 | 1.8e-33 |
| ABP57745.1 | DepA | BGC0000993 | FK228\_biosynthetic\_gene\_cluster | 27 | 26.2314540059 | 143 | 1.8e-33 |
| BAO66530.1 | nonribosomal\_peptide\_synthase | BGC0000042 | Cremimycin\_biosynthetic\_gene\_cluster | 28 | 25.7566765579 | 143 | 1.8e-33 |
| EFL02193.1 | amino\_acid\_adenylation\_domain-containing\_protein | BGC0000996 | Frontalamides\_biosynthetic\_gene\_cluster | 24 | 44.9258160237 | 143 | 2.4e-33 |
| ADH04641.1 | TgaC | BGC0001051 | Thuggacin\_biosynthetic\_gene\_cluster | 27 | 27.3590504451 | 143 | 3.1e-33 |
| ADH04660.1 | TugD | BGC0001342 | Thuggacin\_biosynthetic\_gene\_cluster | 26 | 28.5459940653 | 142 | 5.2e-33 |
| AFR69331.1 | nonribosomal\_peptide\_synthetase\_SpiA | BGC0001045 | Spiruchostatin\_biosynthetic\_gene\_cluster | 25 | 36.9139465875 | 141 | 6.8e-33 |
| CAJ76292.1 | putative\_non-ribosomal\_peptide\_synthase | BGC0000972 | Colibactin\_biosynthetic\_gene\_cluster | 27 | 32.2255192878 | 141 | 6.8e-33 |
| ABC39418.1 | dihydroaeruginoic\_acid\_synthetase | BGC0000964 | Burkholdac\_biosynthetic\_gene\_cluster | 28 | 26.646884273 | 141 | 6.8e-33 |
| AAW03330.1 | CtaG | BGC0000982 | Cystothiazole\_A\_biosynthetic\_gene\_cluster | 30 | 23.8575667656 | 141 | 8.9e-33 |
| ACO94489.1 | putative\_AMP-dependent\_acyl-CoA\_synthetase/ligase | BGC0000097 | ML-449\_biosynthetic\_gene\_cluster | 27 | 31.2759643917 | 141 | 1.2e-32 |
| ABV56594.1 | KtzN | BGC0000378 | Kutznerides\_biosynthetic\_gene\_cluster | 27 | 30.8605341246 | 140 | 1.5e-32 |
| BAO66533.1 | nonribosomal\_peptide\_synthase | BGC0000042 | Cremimycin\_biosynthetic\_gene\_cluster | 27 | 32.2255192878 | 138 | 5.8e-32 |
| AKD43757.1 | HerL | BGC0001349 | Heronamide\_biosynthetic\_gene\_cluster | 27 | 25.4599406528 | 138 | 5.8e-32 |
| ACO94461.1 | putative\_AMP-dependent\_acyl-CoA\_synthetase/ligase | BGC0000029 | BE-14106\_biosynthetic\_gene\_cluster | 27 | 31.2759643917 | 137 | 1.7e-31 |
| BAL15726.1 | NRPS | BGC0000432 | Streptothricin\_biosynthetic\_gene\_cluster | 25 | 35.9050445104 | 136 | 2.2e-31 |
| ABW71852.1 | nonribosomal\_peptide\_synthetase | BGC0000303 | Anthramycin\_biosynthetic\_gene\_cluster | 27 | 29.6735905045 | 136 | 2.2e-31 |
| AAC83657.1 | pyochelin\_synthetase | BGC0000412 | Pyochelin\_biosynthetic\_gene\_cluster | 25 | 33.471810089 | 136 | 2.9e-31 |
| CAD89778.1 | MelG\_protein | BGC0001009 | Melithiazol\_biosynthetic\_gene\_cluster | 30 | 23.8575667656 | 136 | 3.8e-31 |
| CAJ87590.1 | putative\_peptide\_synthase | BGC0001055 | Yersiniabactin\_biosynthetic\_gene\_cluster | 26 | 27.4777448071 | 136 | 3.8e-31 |
| AGI61652.1 | long\_chain\_fatty\_acid\_CoA\_ligase | BGC0000095\_c2 | Micromonolactam\_biosynthetic\_gene\_cluster | 27 | 33.412462908 | 135 | 6.4e-31 |
| CAQ34914.1 | putative\_L-prolyl\_AMP-ligase | BGC0000986 | Dkxanthene\_biosynthetic\_gene\_cluster | 26 | 32.0474777448 | 135 | 6.4e-31 |
| ABP55217.1 | AMP-dependent\_synthetase\_and\_ligase | BGC0000142 | Salinilactam\_biosynthetic\_gene\_cluster | 26 | 31.9881305638 | 134 | 1.4e-30 |
| BAP34718.1 | AMP-dependent\_synthetase\_and\_ligase | BGC0000078 | Incednine\_biosynthetic\_gene\_cluster | 27 | 32.9970326409 | 134 | 1.4e-30 |
| AGC65516.1 | TtbA | BGC0001050 | Thalassospiramide\_biosynthetic\_gene\_cluster | 22 | 71.6320474777 | 134 | 1.4e-30 |
| AKD43754.1 | HerJ | BGC0001349 | Heronamide\_biosynthetic\_gene\_cluster | 26 | 30.5044510386 | 134 | 1.4e-30 |
| CAJ34370.1 | NRPS\_protein | BGC0000445 | Thiocoraline\_biosynthetic\_gene\_cluster | 31 | 24.3916913947 | 133 | 1.9e-30 |
| ABC36203.1 | thiotemplate\_mechanism\_natural\_product\_synthetase | BGC0001102 | Malleilactone\_biosynthetic\_gene\_cluster | 25 | 37.5667655786 | 133 | 2.4e-30 |
| BAD08370.1 | non-ribosomal\_peptide\_synthetase | BGC0000167 | Vicenistatin\_biosynthetic\_gene\_cluster | 26 | 32.9970326409 | 133 | 3.2e-30 |
| BAR73008.1 | putative\_ATP-dependent\_b-aminoacyl-ACP\_synthetase | BGC0001194 | Hitachimycin\_biosynthetic\_gene\_cluster | 25 | 31.3946587537 | 132 | 5.4e-30 |
| BAP34707.1 | AMP-dependent\_synthetase\_and\_ligase | BGC0000078 | Incednine\_biosynthetic\_gene\_cluster | 26 | 32.2848664688 | 130 | 2.1e-29 |
| ALA09365.1 | AMP-dependent\_synthetase\_and\_ligase | BGC0001303 | Lobosamide\_biosynthetic\_gene\_cluster | 25 | 31.9881305638 | 128 | 6e-29 |
| BAL15718.1 | NRPS | BGC0000432 | Streptothricin\_biosynthetic\_gene\_cluster | 27 | 26.587537092 | 128 | 6e-29 |
| AEA29643.1 | putative\_nonribosomal\_peptide\_synthetase | BGC0000409 | Porothramycin\_biosynthetic\_gene\_cluster | 26 | 30.6231454006 | 126 | 3.9e-28 |
| AKC54422.1 | fumosorinone\_biosynthesis\_polyketide\_synthase | BGC0001218 | Fumosorinone\_biosynthetic\_gene\_cluster | 27 | 27.3590504451 | 125 | 6.6e-28 |
| AAF19815.1 | MtaG | BGC0001024 | Myxothiazol\_biosynthetic\_gene\_cluster | 30 | 23.1454005935 | 124 | 1.1e-27 |
| ABS90473.1 | NRPS | BGC0001106 | Oxazolomycin\_biosynthetic\_gene\_cluster | 24 | 40.6528189911 | 122 | 4.3e-27 |
| CDG12865.1 | non-ribosomal\_peptide\_synthetase | BGC0001415 | Althiomycin\_biosynthetic\_gene\_cluster | 23 | 32.7002967359 | 122 | 4.3e-27 |
| CCB53264.1 | non-ribosomal\_peptide\_synthetase | BGC0001393 | Lugdunin\_biosynthetic\_gene\_cluster | 23 | 36.2611275964 | 115 | 6.9e-25 |
| BAC87906.1 | probable\_acinetobactin\_biosynthesis\_protein | BGC0000294 | Acinetobactin\_biosynthetic\_gene\_cluster | 26 | 25.7566765579 | 109 | 4.9e-23 |
| AEO14743.1 | NdaA | BGC0000396 | Nodularin\_biosynthetic\_gene\_cluster | 24 | 31.8100890208 | 108 | 8.4e-23 |
| AFR69337.1 | nonribosomal\_peptide\_synthetase\_SpiE2 | BGC0001045 | Spiruchostatin\_biosynthetic\_gene\_cluster | 25 | 22.6706231454 | 99 | 3.9e-20 |
| ABA59547.1 | Vlm1 | BGC0000453 | Valinomycin\_biosynthetic\_gene\_cluster | 24 | 27.4777448071 | 94 | 1.6e-18 |
| ADI58632.1 | AsuB1 | BGC0000187 | Asukamycin\_biosynthetic\_gene\_cluster | 22 | 27.9525222552 | 75 | 6e-13 |
| ALE27504.1 | 3-HPA:AMP\_ligase | BGC0001292 | A33853\_biosynthetic\_gene\_cluster | 23 | 23.6795252226 | 74 | 1e-12 |
| ADC79613.1 | BafX | BGC0000028 | Bafilomycin\_biosynthetic\_gene\_cluster | 22 | 25.9940652819 | 72 | 5.1e-12 |
| AIS24844.1 | Dst22 | BGC0001147 | Distamycin\_biosynthetic\_gene\_cluster | 23 | 32.1068249258 | 69 | 3.3e-11 |
| CAJ88630.1 | putative\_acyl-CoA\_synthetase | BGC0000327 | Congocidine\_biosynthetic\_gene\_cluster | 22 | 26.706231454 | 68 | 7.4e-11 |
| WP\_010639243.1 | 2-succinylbenzoate--CoA\_ligase | BGC0000958 | Antimycin\_biosynthetic\_gene\_cluster | 22 | 21.5430267062 | 52 | 7.1e-06 |
